# Chemo-Enzymatic Synthesis of Viscoelastic Pseudopeptidoglycan Networks

**DOI:** 10.1021/acs.bioconjchem.5c00014

**Published:** 2025-06-24

**Authors:** Philipp Loibner, David Bučak-Gasser, Katharina Schober, Tobias Steindorfer, Monika Brandtner, Tobias Dorn, Tanja Wrodnigg, Dmytro Neshchadin, Georg Gescheidt-Demner, Matej Bračič, Florian Lackner, Tamilselvan Mohan, Karin Stana Kleinschek, Rupert Kargl

**Affiliations:** † Institute of Chemistry and Technology of Biobased Systems, IBIOSYS, 27253 Graz University of Technology, Stremayrgasse 9, 8010 Graz, Austria; ‡ Institute of Physical and Theoretical Chemistry, 27253 Graz University of Technology, 8010 Graz, Austria; § Institute of Engineering Materials and Design, 112807 University of Maribor, Maribor 2000, Slovenia

## Abstract

Bacterial peptidoglycans (PGs) are essential targets for antibiotics and immune cells. Chemical methods to reproduce PGs semisynthetically are tedious and wasteful. In this work, we describe a new approach to form pseudo-PGs (PPGs) using the protease papain and custom-made peptides conjugated to a glycan. The kinetics of formation is monitored by rheology and ^1^H NMR. Viscoelastic gels of controlled strength are formed, depending on the temperature and the number of peptide bridges between the glycan chains. We propose that the new method has a high impact on biomaterials research, since it could be used to deliver peptides, test antibiotic efficacy, or investigate human immune cell response.

## Graphical abstract

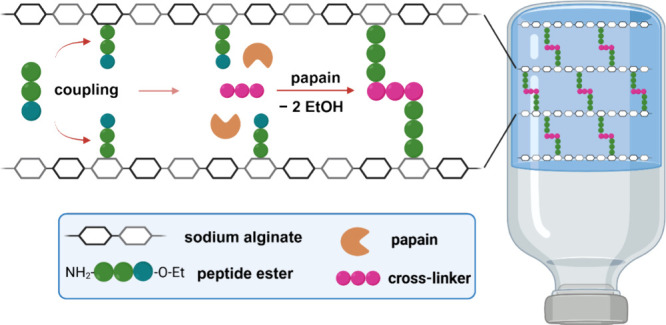

## Introduction

The peptidoglycans (PGs) of bacterial cell walls are supramolecular networks of cross-linked, peptide-grafted glycan chains.^1,2^ PG biosynthesis is an essential target for antibiotics, and the human immune system can recognize the building blocks and the resulting structural motifs.^3^ During biosynthesis, amino acids and short peptides are attached via the N-terminal to the *N-*Acetyl muramic acid (MurNAc) unit of the glycan through amide bonds catalyzed by Mur ligases.^1^ The bridges between the protruding peptides of adjacent chains are built by the transpeptidase domain of penicillin-binding proteins.^1,4^ PGs contribute to the resistance of the bacterial cell wall, and the mechanical properties of these hydrogel networks are determined by the glucan, its length, orientational ordering, and by the number and type of bridging peptide structures.^1,2,5^

Methods to reproduce these structures in the form of pseudo-PGs (PPGs) are therefore of high interest,^6–13^ and the term is also used for PPGs in some archaea to distinguish them from bacterial PGs.^14^ Semisynthetic PPGs can benefit our understanding of bacterial, archaean, and fungal cell walls and their biosynthesis,^15,16^ can be used to investigate endothelial or immune cellular response,^12,17,18^ and can yield biomaterials for drug delivery or cell culture.^19,20^ Especially for the latter, it is of high interest to imitate the biomechanical and viscoelastic properties of the target tissue,^21^ which is, in many cases, strain stiffening due to cross-linked peptide residues.^22–27^ PPGs are, therefore, potential candidates to create strain-stiffening gels.

Currently, peptides previously synthesized by solid phase synthesis (SPPS) are exclusively conjugated chemically to glycans using conventional peptide coupling agents,^19^ protecting group strategies,^28^ or click chemistry.^29^ Cross-linking into pseudo-PG gel networks is then accomplished chemically with conventional reactants,^30^ click reactions,^31^ or by using the enzyme transglutaminase, forming isopeptide bonds.^32,33^ Even though proteases are known to form α-peptide bonds from C-terminal amino acid esters, this has not been utilized to produce defined pseudo-PG networks.^32–34^

We herein therefore report about a chemo-enzymatic method that allows for the formation of short peptide bridges between alginate glycan chains (**1**), leading to defined pseudo-PG networks as shown in Figure 1. In this approach, tripeptides with C-terminal ester groups (**2**) are chemically grafted to the glycan via amide bonds using (4-(4,6-dimethoxy-1,3,5-triazin-2-yl)-4-methyl-morpholinium chloride), giving **3**. This is followed by imitating the bacterial transpeptidase reaction with a cysteine protease-catalyzed aminolysis of the ester bonds, using previously synthesized, glycine-terminal cross-linkers (**6b, 8b**) that were fully characterized by NMR, FTIR, elemental analysis, and potentiometric charge titration. The enzymatic gelation processes are monitored via concentration, time- and temperature-dependent oscillatory shear rheology, yielding information about the kinetics of gelation and stability of the pseudo-PGs. The results are supported by kinetic NMR experiments measuring the papain-catalyzed ester cleavage. The aim of this study is to measure and prove that pseudopeptidoglycans can be produced by a combination of chemical synthesis and protease catalysis, and to determine viscoelastic properties and product formation kinetics in situ.

**Figure 1:**
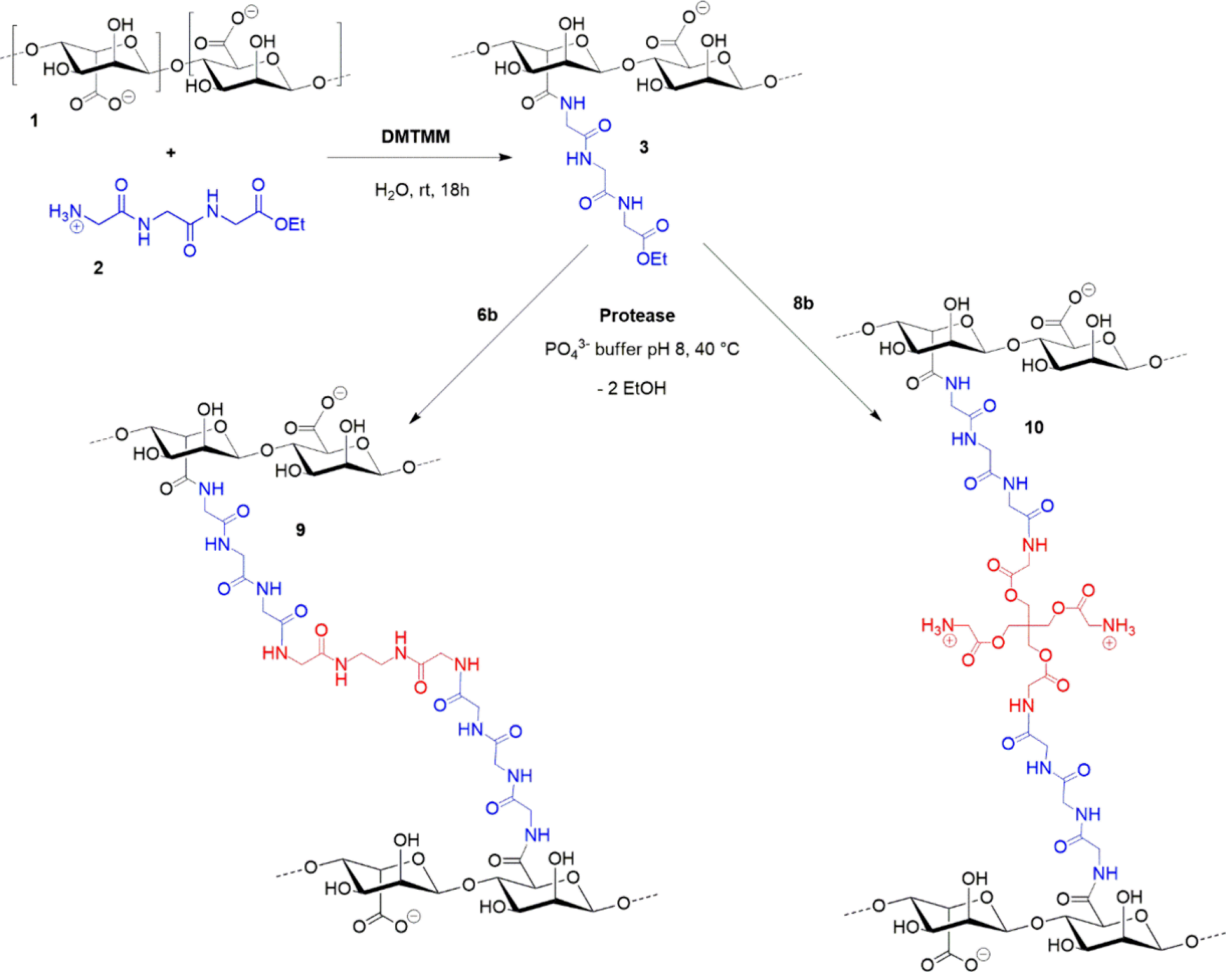
Conjugation of sodium alginate (**1**) with a C-terminal peptide ethyl ester (**2**) and subsequent protease catalyzed cross-linking with glycine N-terminal cross-linkers (**6b/8b**) to form pseudopeptidoglycan (PPG) gel networks (**9/10**).

## Results and Discussion

### Selection of the Papain Peptide Substrate

Papain has a well-known preference for amino acid esters with hydrophobic residues as acyl donors. The ester group, opposed to the free carboxylate, functions as an activated acyl with increased binding affinity to the nucleophilic cysteine in the catalytic triad.^35^ In accordance with this trend, the substrate preference of the ethyl over the methyl ester was found in preliminary studies (Table S3), where oligomerization of glycine peptide ethyl esters resulted in a higher yield of isolable solids than in the case of the methyl ester. Alginate peptide conjugate **3** (Figure 1) was, therefore, synthesized as a precursor to pseudo-PGs.

### Alginate Peptide Conjugates

#### ATR-IR, ^1^H NMR Spectroscopy, and Gel Permeation Chromatography

The ATR-IR spectra of alginate **1**, peptide ethyl ester **2** and the alginate peptide conjugate **3** show the characteristic stretching vibrations of the ethyl ester (C=O, *ṽ*: 1740 cm^–1^), amide I (C=O, *ṽ*: 1690 cm^–1^), free carboxylate (C=O, *ṽ*: 1650 cm^–1^), and amide II (NH, *ṽ*: 1560 cm^–1^), proving the presence of the intact ester (Figure 2A). The ^1^H NMR spectrum (Figure 2B) of the alginate peptide conjugate **3** shows the characteristic peaks of the ethyl ester protons (−CH_3_, δ = 1.23 ppm, 3H, t; −CH_2_–, δ = 4.15 ppm, 2H, q) together with the intrachain methylene protons of the peptide (−CH_2_–, δ = 3.96 ppm, 2H, s; δ = 3.99 ppm, 2H, s). These signals do not shift significantly upon conjugation. Contrarily, the signal of the N-terminal methylene protons, clearly visible in the unbound peptide (−CH_2_–, δ = 3.84 ppm, 2H, s), shifts toward lower fields upon conjugation, indirectly confirming a covalent attachment to the alginate through an amide bond. Minor amounts of unbound peptide seem to be present. Methyl (−CH_3_, δ = 1.15 ppm, 3H, t) and methylene protons (−CH_2_–, δ = 3.55 ppm, 2H, q) of ethanol are visible in the conjugate, stemming either from the isolation/precipitation, or the partial hydrolysis of the peptide during the conjugation, workup or measurement (see also Figure 7). Amine or amide protons could not be detected via ^1^H NMR, since rapid exchange annihilates the respective signals in the solvent used (D_2_O). Due to the broad and overlapping NMR signals of alginate, it is not possible to deduce information about the preferential amidation of either L-guluronic or D-mannuronic units of the polymer.^36^ In our opinion, analysis of the substitution pattern would require enzymatic depolymerization of the peptide conjugates into oligo- and monomers, followed by high-resolution NMR or chromatographic separation combined with mass spectrometry.

**Figure 2:**
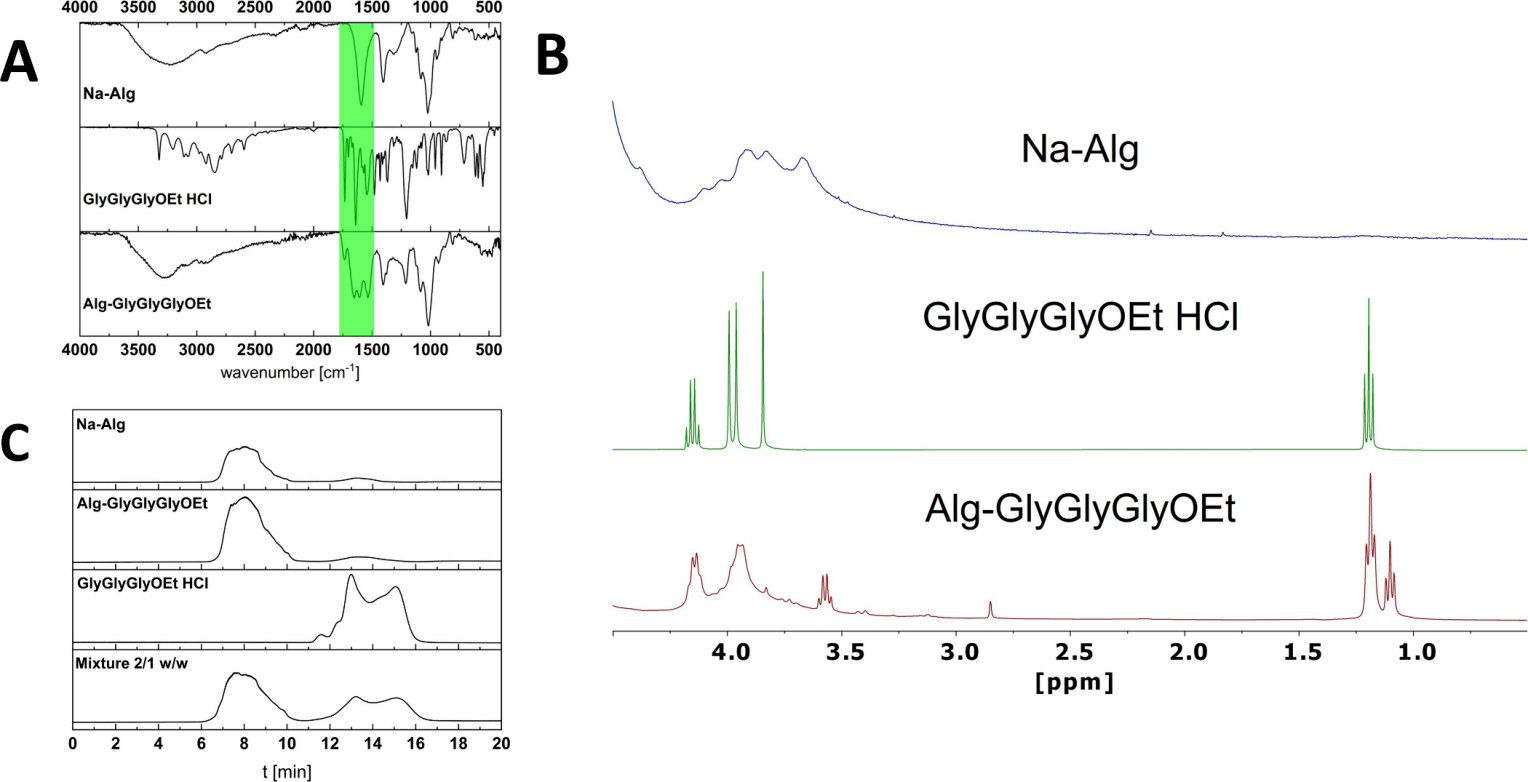
(A) ATR-IR spectra; (B) ^1^H NMR spectrum (D_2_O); (C) GPC-RI of the modified alginate Alg-GlyGlyGlyOEt **3**, compared to unmodified Na-Alg **1**, and GlyGlyGlyOEt **2**. A mixture of Alg-GlyGlyGlyOEt **3** and GlyGlyGlyOEt **2** (2/1 w/w) is shown in (C) for comparison.

Further evidence for covalent conjugation to the alginate is obtained by comparing the respective gel permeation chromatograms (GPC-RI) of reactants and products (Figure 2C). The hydrodynamic radii of the fractions of alginate (retention times 7.8, 13.3 min) are not changed significantly with the conjugation. The shape and peak area of the conjugate indicate that only minor amounts of unbound peptide are present. The chromatogram of a solution (mixture 2/1 w/w) of alginate peptide conjugate (**3**), spiked with free peptide ethyl ester (**2**), confirms the separability of both components under the chromatographic conditions employed.

### Small Molecular Cross-Linking Agents

The protease papain prefers amino acid derivatives as substrates for amide formation.^37^ Uyama et al. have shown with their study on papain-catalyzed oligomerization of glutamic acid diethyl ester that the enzyme has a strong regioselective preference for forming amide bonds on the α-position rather than the γ-position.^38^ Thus, glycine-derived cross-linking agents (compounds **6b** Figure S1, and **8b** Figure S8) were synthesized, which function as small N-terminal linker molecules between the modified polymer chains. Compound **6b** (2GlyEDA) represents a bifunctional cross-linking agent where the glycine moieties are connected via amide bonds, as opposed to compound **8b** (4GlyPE), a tetrafunctional cross-linking agent where glycine moieties are connected via ester bonds. Differences concerning stability toward hydrolysis as well as amine availability for the enzyme are discussed in the sections below.

The molecular structure of the glycine-based cross-linking agents was confirmed via elemental analysis, ^1^H- and ^13^C NMR and infrared spectroscopy (see the Experimental Section and the Supporting Information). The reaction progress was tracked easily by ATR-IR spectroscopy. Distinctive C=O stretching bands corresponding to the protecting carbamate and carboxylate groups of *N*-Boc glycine are visible, but shifted after the amidation (2BocGlyEDA, compound **6a,** (Figure 3, left). Boc-cleavage results in only one band in this region, corresponding to the final amide product, C=O (Figure 3, *bottom*). Similar results are obtained for compound 4GlyPE HCl (**8b**).

**Figure 3:**
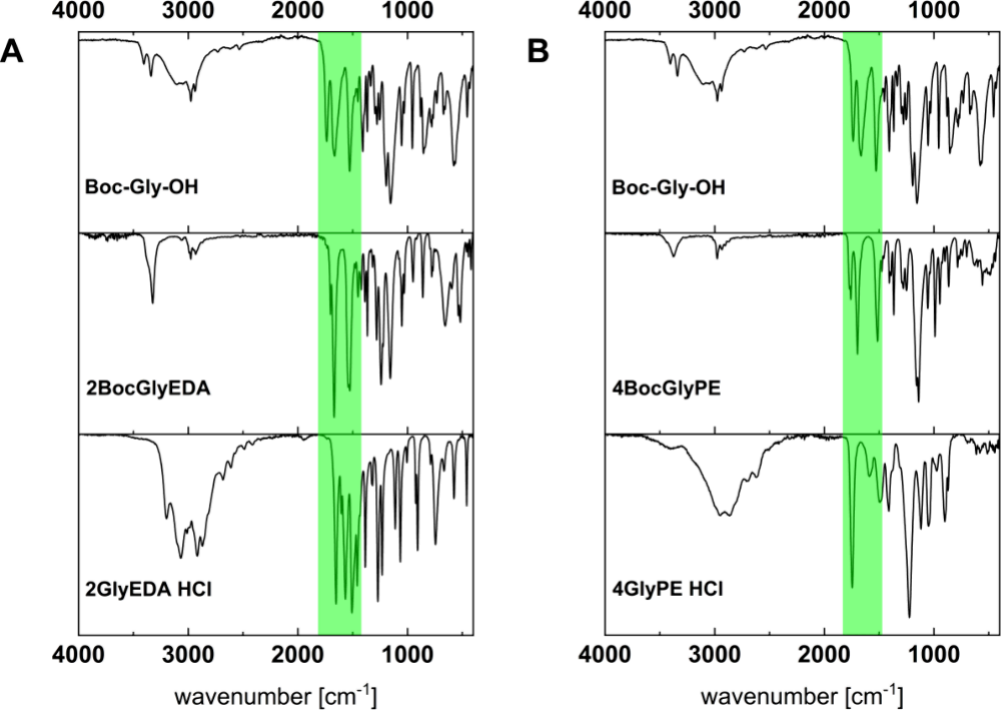
ATR-IR spectra of (A) 2GlyEDA HCl (**6b**, bottom left) and (B) 4GlyPE HCl (**8b**, bottom right) compared to their N-protected analogues (**6a**, **8a**, middle) and Boc-Gly-OH (top); the characteristic C=O stretching bands between 1500 and 1700 cm^–1^ allow for tracking of the respective reaction steps.

### Charge and Stability Considerations

Capping off the free carboxylates of the alginate with the peptide results in a reduction of negative charge on the polymer chain, and the presence of only minor amounts of N-terminal amines from noncovalently bound peptide (p*K*_a_: 7.5–9) as detected by potentiometric charge titrations (Figure 4A, B). Product **3** (Alg-GlyGlyGlyOEt) has a lower charge per mass compared to unmodified reactant **1** (Na-Alg). This allows for an estimation of the degree of substitution (DS) using eq 1 (see the Experimental Section). An average DS of 0.48 was calculated (*n* = 3). It must be noted that a higher DS limits the water solubility of the polymer due to the resulting lack of free carboxylates and potential aggregation of the peptide substituent. To limit the DS, the molar ratio of peptide to uronic acid in alginate was set to 0.5:1 in the conjugation reaction. Partial hydrolysis of the peptide ethyl ester during titration is seen in Figure 4B, with an increase in the amount of carboxylate groups when forward (pH 2 → 11) and back (pH 11 → 2) titration curves are compared. The hydrolytic cleavage of the ethyl ester of the peptide conjugate (**3**) at higher pH values (>9) is not problematic since papain catalysis for cross-linking is performed at neutral to slightly acidic pH values. (see also Figure 7). The amide bond between the peptide and the alginate is stable during titration since only minor amounts of free amine (p*K*_a_: 7.5–9) could be detected in the forward and backward curves.

**Figure 4:**
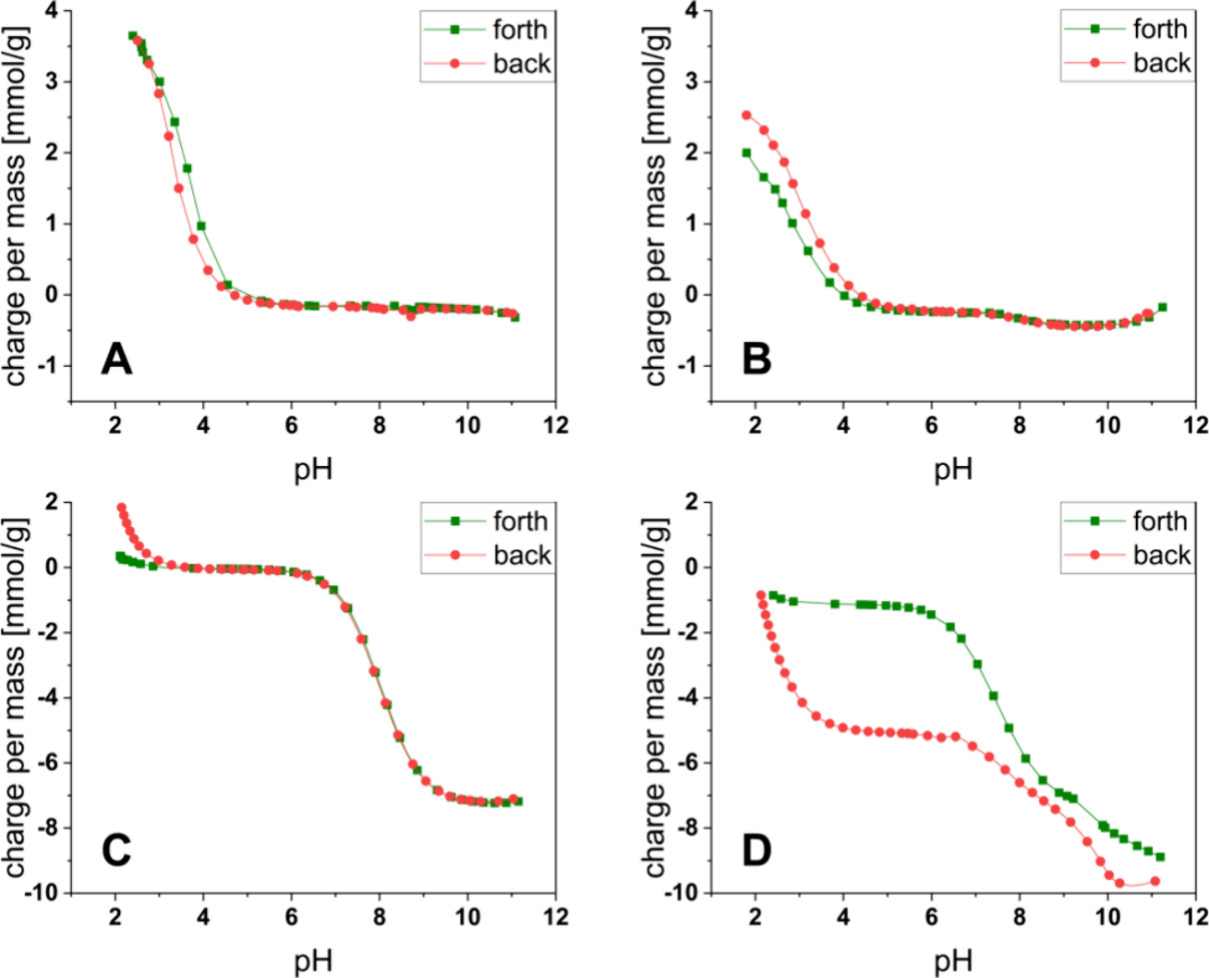
Charge titration measurements; (A) sodium alginate (Na-Alg, (**1**)); (B) alginate peptide conjugate (Alg-GlyGlyGlyOEt, (**3**)); (C) small molecular bifunctional cross-linking agent 2GlyEDA HCl (**6b**); and (D) small molecular tetrafunctional cross-linking agent 4GlyPE HCl (**8b**).

The titration curves of the small molecular cross-linking agents show a significant difference in the pH value-dependent stability (Figure 4C, D). The ester bonds in 4GlyPE HCl (**8b**) hydrolyze under base catalysis at pH > 9, while the hydrolysis of the amide bonds in 2GlyEDA HCl (**6a**) is less pronounced but visible by the increased carboxylate content (p*K*_a_ 2.3) in the back-titration from pH 11 to pH 2. Controlled ester or amide cleavage with the resulting decomposition of the final biomaterial could be a desired property of gels.

### Protease-Catalyzed Cross-Linking

Considerations on reaction conditions and substrates: papain, as found in nature, is known for its strong proteolytic activity.^39,40^ In order to sufficiently shift the equilibrium from hydrolysis to amide formation, adjustment of the reaction conditions and choice of substrates are crucial. A detailed study centered around the interplay between the different reaction conditions and substrates was performed by Li et al.^41^ For biomaterial gels, it is preferred to operate near physiological conditions due to partial evaporation of water as well as possible cell decline in biological systems. Thus, enzymatic reactions in this work were performed mostly at 40 °C, in accordance with previously reported studies.^42,43^

Li et al. observed a significant decrease in the pH value due to the release of HCl/carboxylic acid during papain-catalyzed oligomerization of glutamic acid diethyl ester hydrochloride.^41^ This could also be confirmed for the cross-linking agents of this work. To maintain the pH value within the enzyme′s optimal range of pH 5–8 during the cross-linking reaction and to avoid competing acid or base-catalyzed hydrolysis, a buffering system with a slightly basic initial pH value and high buffering capacity is necessary. This can be compromised by the limited solubility of the alginate peptide conjugate in aqueous solutions of higher ionic strengths. Therefore, cross-linking experiments were conducted in 0.5 M phosphate buffer at pH 8, by first dissolving the modified alginate in half the final volume of deionized water before adding the same volume of 1 M phosphate buffer.

### Rheology

Preliminary cross-linking experiments were conducted with 2 wt % of **3** (alginate peptide conjugate), 10 mg/mL papain, and 10 mM of the respective cross-linker molecule at 40 °C. Alginate conjugates with glycine ethyl ester or glycylglycine ethyl ester, synthesized by the same method as compound **3**, did not lead to cross-linked gels, demonstrating the lower accessibility of the shorter substituent on the alginate chain to the enzyme (data not shown). Gelation only occurred when the glycine-derived cross-linking agents were used and not when l-lysine or ethylenediamine were used. Hydrogels could also be generated by using Dulbecco’s Modified Eagle’s Medium (DMEM) with low glucose content as a solvent. The cell medium’s sophisticated buffering system is therefore suited for the papain-catalyzed amide formation. An image of the so-formed hydrogel can be seen in the Supporting Information (Figure S21). Gelation processes of solutions containing 2 wt % of the alginate conjugate with varying cross-linking agent concentrations were monitored via time-dependent temperature-controlled oscillatory shear rheology measurements for 2–4 h. The cross-linking agents were compared with respect to gelation time *t*_*G*_, reached equilibrium storage moduli *G′*_eq_, and stability. Subsequently, the temperature dependence of the gelation rate was determined in one system.

### Comparison of the Cross-Linking Agents and Concentration Dependency

4GlyPE HCl (**8b**) results in significantly higher maximum storage moduli *G*′**_max_ at lower gelation times than 2GlyEDA HCl (**6b**) (Table 1). This is due to the higher molar amounts of amine available, accompanied by the ability to form three-dimensionally cross-linked structures, where more than two polymer chains are bridged. The ester linkage between the glycine moieties, however, makes the gels more susceptible to hydrolysis. Furthermore, papain can remain active in the gel and start to decompose bridges due to a shift in equilibrium driven by substrate availability. Both effects are assumed to contribute to the notable loss of the gel's firmness after some time, as seen in Figure 5A–C. The time threshold until hydrolytic decomposition *t*_*hyd*_ and the gelation time show a clear concentration dependency. At higher concentrations, gelation takes longer and remains stable for a longer period. At higher concentrations, the gelation takes longer and remains stable for a longer period. An explanation for this can be that binding of the cross-linking agent on one side of the alginate peptide chain is kinetically and sterically favored at high concentrations, consuming more peptide esters without cross-linking to happen. After gelation, hydrolytic decomposition is slower due to the higher number of overall cross-links. In any case, a different gel network structure can be assumed for all three concentrations.

**Figure 5:**
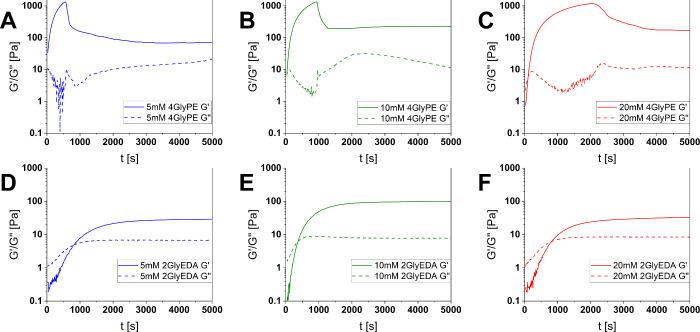
Time-dependent oscillatory shear rheology measurements using 2GlyEDA (**6b**, above) and 4GlyPE (**8b**, below) at concentrations 5 mM (left), 10 mM (middle) and 20 mM (right) for enzymatic cross-linking of (**3**); the ester bound cross-linking agent shows hydrolytic decomposition after a concentration dependent time threshold.

**Table 1:** Descriptive Parameters for the Monitored Gelation Processes

	2GlyEDA HCl (6b)		4GlyPE HCl (8b)			
	*t*_G_ **[s]**	*G*′**_eq_ **[Pa]**	*t*_G_ **[s]**	*G′*_eq_ **[Pa]**	*G*′**_max_ **[Pa]**	*t*_hyd_ **[s]**
5 mM	794	30.1	<30^a^	108.2	1350.7	555
10 mM	368	100.4	32	216.3	1336.8	922
20 mM	820	35.3	105	164.9	1183.9	2035

a Gelation with 4GlyPE HCl at 5 mM was already finished between application of the sample and onset of the measurement.

Similar thoughts can be applied to 2GlyEDA HCl (**6b**). Figure 5D–F shows the existence of an optimal concentration with a short gelation time and high storage moduli. At low concentrations, substrate availability is limited, while at higher concentrations, favorable binding on one side of the alginate chain delays the gelation process. Gelation at higher concentrations is presumably further restricted by the limited mobility of the enzyme within the gel. At all concentrations, the gels show higher resistance against hydrolysis, supporting the results from charge titrations.

### Temperature Dependency

The effect of temperature on the activity of papain and the gelation rates was determined by using 10 mM 2GlyEDA HCl (Figure 6). The gelation time *t*_G_ increases drastically with decreasing temperature, following an approximately exponential correlation between temperature and enzymatic activity (Table 2).

**Figure 6:**
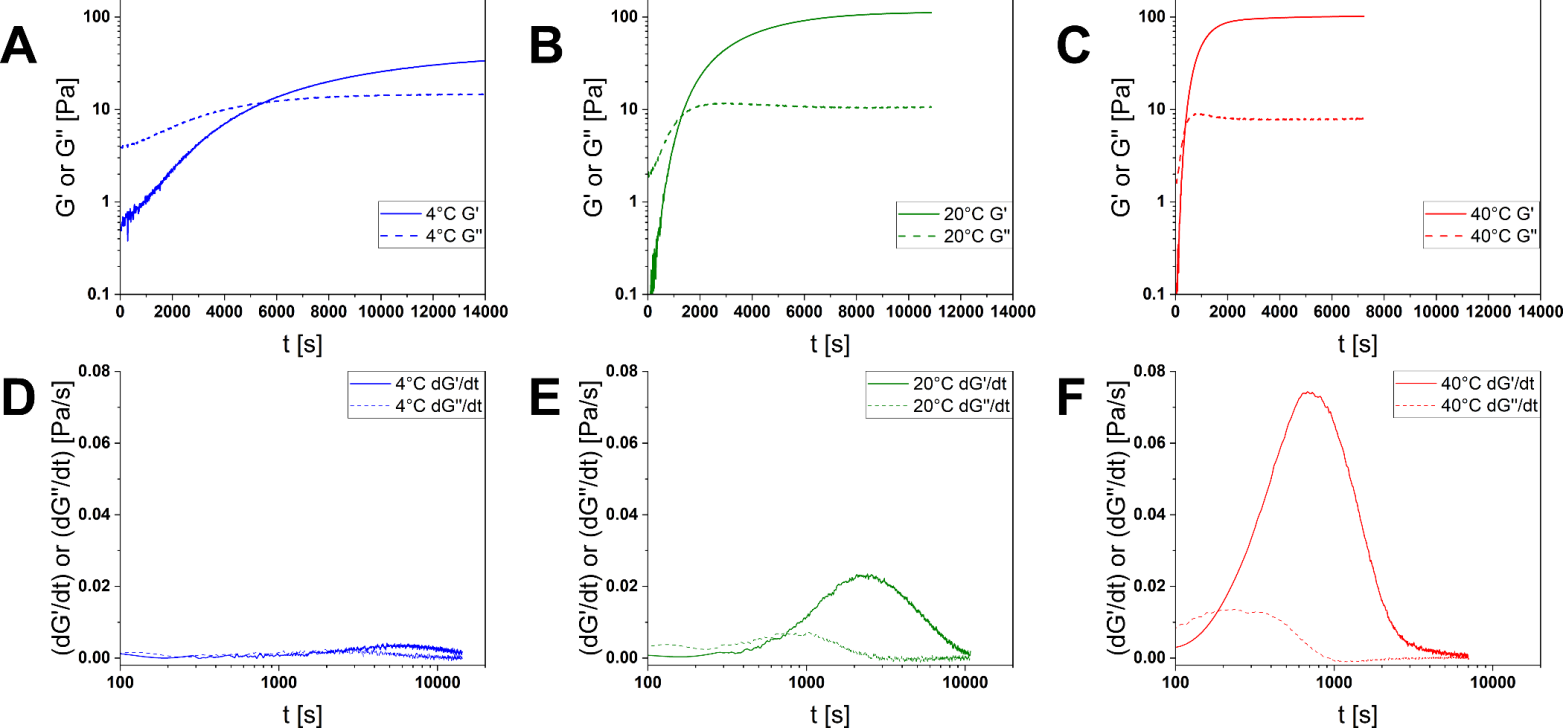
(A–C) Time-dependent oscillatory shear rheology measurements using 10 mM 2GlyEDA (**6b**) as a cross-linking agent *for* (**3**) at 4 °C, 20 °C and 40 °C (above; from left to right) (D–F) with their respective first derivatives of storage and loss moduli on a logarithmic time scale (below); temperature decrease influences the gelation rate due to reduced enzymatic activity.

**Table 2:** Gelation Time and Maximal Gelation Rate Using 10 mM 2GlyEDA HCl (**6b**) at Different Temperatures

	*t*_G_ [s]	(d*G′*/d*t*)_max_ [Pa/s]
40 °C	368	0.074
20 °C	1315	0.023
4 °C	5507	0.004

As a metric for the gelation activity of papain, the first derivative of the storage modulus over time d*G*′**/d*t* was calculated (Figure 6, below). Comparing the maximal gelation rates (d*G*′**/d*t*)_max_ gives a broader picture of the gelation capability of the enzyme at different temperatures. The calculated values follow a similar exponential pattern to the gelation times. Complete inhibition of gelation might demand other strategies, such as protease inhibitors, but a reduced temperature is a strong supporting factor.

### Kinetic Study via ^1^H NMR

Differences in the enzyme-catalyzed reaction rates for a 2 wt % solution of alginate peptide conjugate **3** were obtained via kinetic ^1^H NMR experiments. Peptide ester consumption and ethyl alcohol release were measured over time with and without the presence of glycine. Twenty mM glycine instead of 2GlyEDA HCl as the nucleophile was used to avoid cross-linking and an increase in viscosity during measurements. Still, care must be taken when interpreting the data since the p*K*_a_ value for glycine (p*K*_a_ 9.6) and 2 GlyEDA (**6b**, p*K*_a_ 8.1) differ, and with them, most likely also nucleophilicity. Spectra were recorded for 2–3 h with gaps of 190 s at ambient temperature. For quantification, the triplets at 1.23 and 1.15 ppm, corresponding to the terminal CH_3_ of the ethyl ester and the free ethyl alcohol, were integrated (Figure 7, left). Plotting the progression of the relative concentrations in dependence on time gave exponential correlations in the form of *y* = exp­(−*k*′*t*) or *y* = 1 – exp­(−*k*′*t*) corresponding to a pseudo-first order reaction (PFO), approximated for low substrate concentrations (Figure 6, right).

**Figure 7:**
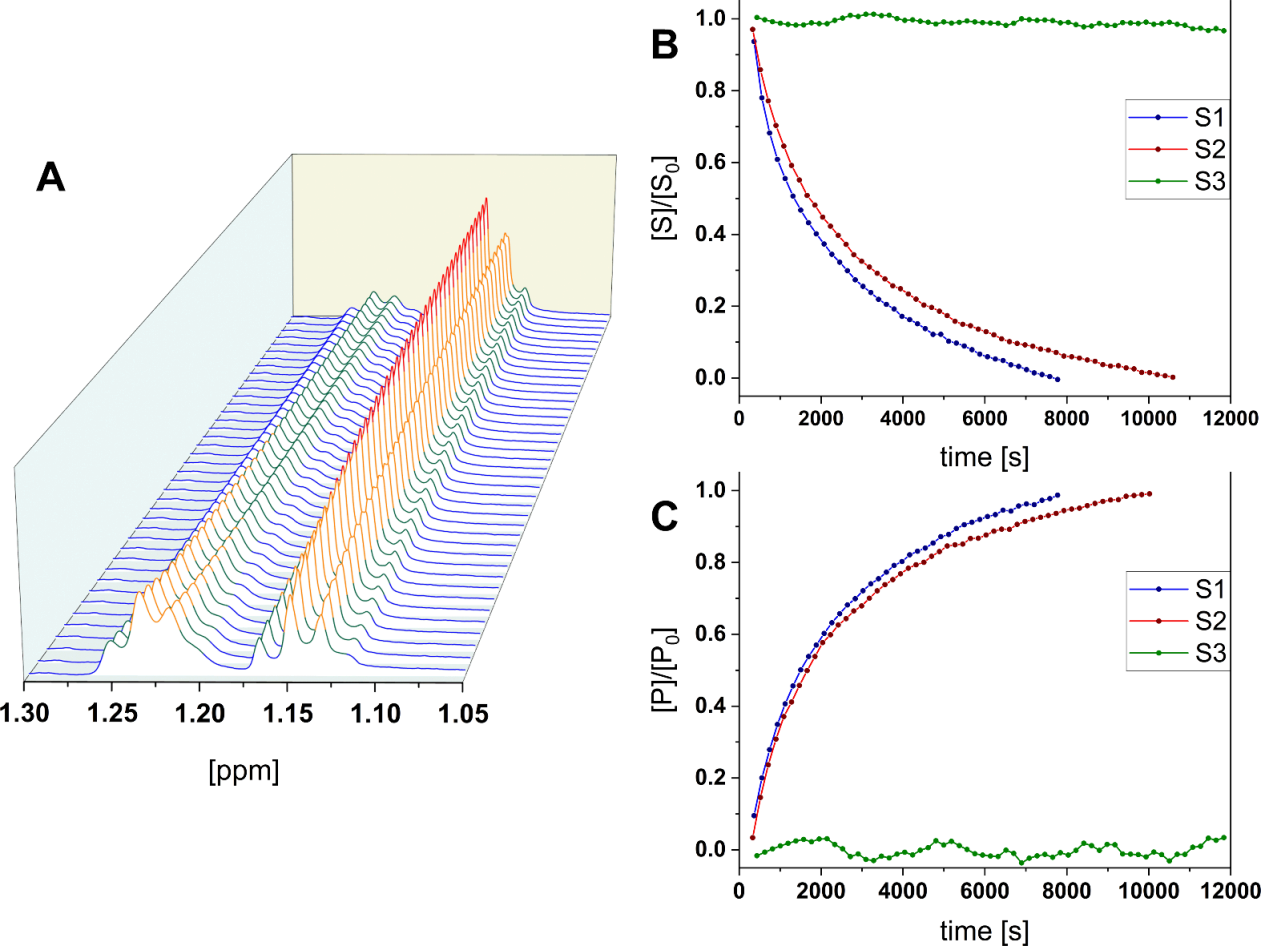
Kinetic contemplation of ester hydrolysis. left: aligned spectra of **S1** monitored for 2 h, decrease of ester concentration can be determined at 1.23 ppm and alcohol release at 1.15 ppm; right: time-resolved progress of relative concentrations of the ethyl ester ([S], above) and ethanol ([P], below) for **S1**–**S3**, fitting a first order correlation gives the respective rate constants *k′*.

Three different systems were monitored, which are summarized in Table 3. Release of ethyl alcohol from alginate peptide conjugate **3** is only slightly faster if glycine (**S2**) instead of water/OH^–^ (**S1**) is offered as a nucleophile. This is attributed to the fact that aminolysis of the covalent enzyme alginate-conjugate thioester complex is faster than its hydrolysis, regenerating the enzyme quicker, and making it available for the peptide ester hydrolysis. The orders of magnitude of the reaction rates are very similar to those for the rheological experiments. Ethyl alcohol release without the presence of papain (**S3**) does not occur at the pH value investigated; the calculated relative integrals fluctuate only around the starting concentrations. Ethanol present at measurement onset originates mostly from the workup of modified alginate (Figure 2).

**Table 3:** Kinetic Parameters of the Ester Hydrolysis with the Exclusion of Either Glycine or Papain

				ester consumption			alcohol release		
	mod Alg (wt %)	*c (Gly)* [mM]	*c* (*Pap*)[mg mL^–1^]	*R* ^2^	*k′* [s^–1^]	*t*_1/2_[s]	*R* ^2^	*k*′** [s^–1^]	*t*_1/2_[s]
**S1**	2	20	10	0.990	4.7 × 10^–4^	1488	0.996	4.3 × 10^–4^	1608
**S2**	2		10	0.992	3.7 × 10^–4^	1896	0.992	3.8 × 10^–4^	1826
**S3**	2	20							

## Conclusions

A convenient and efficient way to synthesize aqueous pseudopeptidoglycan (PPG) gel networks is presented. A peptide C-terminal ethyl ester with a necessary minimum of three glycine residues is conjugated to uronic acids of alginate through amide bonds, yielding water-soluble glycan peptide derivatives. Glycine-based amide-bound bi-, and ester-bound tetravalent cross-linkers are chemically synthesized, isolated, and characterized. The bivalent cross-linkers are more stable against hydrolytic degradation than the ester-based tetravalent substances. The protease papain is used to build amide bridges between the protruding peptide grafts on the alginate chain, yielding cross-linked, stable PPG gels with adjustable network architectures. The curing rate of the networks decreases with increasing concentration of the tetravalent cross-linker but shows a maximum for a median concentration of the bivalent cross-linker. A decreased temperature reduces the curing rate. The kinetics of the protease-catalyzed release of ethyl alcohol from the alginate peptide conjugate can be conveniently monitored by ^1^H NMR, demonstrating the stability of the ester without enzyme and the efficiency of enzymatic hydrolysis. Aminolysis of the presumably covalent complex between the enzyme and the alginate peptide conjugate proceeds slightly faster than its hydrolysis. More data must be gathered in 2D NMR or isotope labeling experiments to elucidate the interaction of the enzyme with the glycan peptide conjugate.

The concept can be extended to other biologically and immunologically relevant polysaccharides and peptides or toward real peptidoglycan imitates. For applications, toxicological tests of the individual components and processes are crucial. Potential applications include injectable hydrogels, scaffolds for tissue culture, or drug delivery and materials imitating bacterial cell walls for immunological studies.

## Experimental Section

### Materials

Alginic acid sodium salt from brown algae (Na-Alg, BioReagent); 1,1*′*-carbonyldiimidazole (CDI, reagent grade, caution supplier safety data sheet: acute tox. 4 oral, eye dam. 1, skin corr. 1B); ethylenediamine (EDA, > 99%, caution: acute tox. 3 dermal, acute tox. 4 inhalation, acute tox. 4 oral, aquatic chronic 3, eye dam. 1, flam. liq. 3, respectively sens. 1B, skin corr. 1B, skin sens. 1B); pentaerythritol (PE, 98%); *N-(tert*-Butoxycarbonyl) glycine (Boc-Gly-OH, Novobiochem­(R)); hydrogen chloride solution (4.0 M in dioxane, caution: carc. 1B, eye irrit. 2, flam. liq. 2, - met. corr. 1, skin irrit. 2, STOT SE 3); glycine (ACS reagent, ≥ 98.5%); glycine methyl ester hydrochloride (H-GlyOMe HCl, caution eye dam. 1), glycine ethyl ester hydrochloride (H-GlyOEt HCl, caution eye dam. 1), and glycylglycine methyl ester hydrochloride (H-GlyGlyOMe HCl, caution: acute tox. oral 4, skin corr. 2, serious eye damage/eye irritation 2A, STOT SE 3) and Dulbecco’s Modified Eagle’s Medium (DMEM, low glucose, suitable for cell culture) were purchased from Merck (formerly Sigma-Aldrich), Germany. 4-(4,6-dimethoxy-1,3,5-triazin-2-yl)-4-methyl-morpholinium chloride (DMTMM, 97%, caution: acute tox. 4 oral, skin corr. 1B) was purchased from Carbolution Chemicals GmbH, Germany. 1-Ethyl-3-(3-(dimethylamino)­propyl)­carbodiimide hydrochloride (EDC HCl, 98%, caution: acute tox. 3 dermal, acute tox. 4 oral, aquatic acute 1, aquatic chronic 1, skin irrit. 2, skin sens. 1) was purchased from abcr GmbH, Germany. Glycylglycylglycine ethyl ester hydrochloride (H-GlyGlyGly-OEt HCl, caution: acute tox. oral 4, skin corr. 2, serious eye damage/eye irritation 2A, STOT SE 3) was purchased from BLDpharm, USA. Nanofibrillated cellulose (NFC, 3 wt % suspension in H_2_O, Valida, containing a fungicide) was purchased from Sappi, Netherlands. Papain from *Carica papaya* (>30,000 USP-U/mg) was purchased from Carl Roth GmbH + Co. KG, Germany. Silicon oil (technical, kinematic viscosity: 50 mm^2^ s^–1^, density: 0.96 kg L^–1^) was purchased from VWR Chemicals, Austria. SYLGARD 184 Silicone Elastomer Kit was purchased from DOW AUSTRIA GmbH, Austria.

### Peptide Conjugation to Alginate

The following procedure was derived from Labre et al.^36^ and can be applied similarly to the modification of alginate with other glycine peptide methyl- or ethyl esters with chain length 1–3; 0.3 g (1.4 mmol monomeric units) of Na-Alg was dissolved in 30 mL of H_2_O dest. (1 wt %). DMTMM (0.39 g, 1.4 mmol) was added to the solution, and the mixture was stirred for 10 min. Then, H-GlyGlyGly-OEt HCl (0.18 g, 0.7 mmol) was added, and the solution was stirred for 18 h at rt. The modified alginate was precipitated in 90 mL of EtOH and stirred for 10 min. The mixture was sieved through an unequipped Büchner funnel, and the resulting aggregate was washed with EtOH (3x). The peptide conjugate was purified by dissolving in 30 mL of H_2_O dest. and subsequent repetition of the precipitation procedure. Excess EtOH was removed under reduced pressure. Freeze-drying gave the modified polymer (Alg-GlyGlyGlyOEt, **3**) as a white solid (0.29 g, polymer recovery: 95%). ATR-IR: 3277, 3099, 2938, 1739, 1652, 1608, 1534, 1403, 1213, 1084, 1017 cm^–1^; ^1^H NMR (400 MHz, D_2_O): δ = 4.14 (q, CH_2_), 3.99 (s, CH_2_), 3.95 (s, CH_2_), 3.93 (s, CH_2_), 3.83 (s, CH_2_), 1.19 (t, CH_3_); avg. DS: 0.48

### Hydrogel Preparation

A total of 0.04 g of the modified alginate **3** was dissolved in 1 mL of H_2_O dest. under vigorous stirring. 0.5 mL of a solution containing cross-linking agent (compounds **6b** or **8b**) with a predetermined concentration in 1 M phosphate buffer (pH 8) was added, and the solution was brought to a predetermined temperature. The final cross-linker concentrations in the gel mixture, including the enzyme solution, were set to 5, 10, or 50 mM. To start the gelation process, 0.5 mL of a papain solution (40 mg/mL in 1 M phosphate buffer, pH 8, prepared and tempered directly before addition) was added. For rheological measurements, the solutions were mixed thoroughly with a pipet tip and the viscous mixture was applied on the sample plate of the instrument immediately (applied volume: 0.25 mL).

## Instrumental Methods

### Attenuated Total Reflectance Infrared Spectroscopy (ATR-IR)

IR spectra were measured on an ALPHA-P (Bruker, USA) spectrometer with a scan range of 400–4000 cm^–1^ at a resolution of 4 cm^–1^ (40 scans).

### Nuclear Magnetic Resonance Spectroscopy (NMR)

NMR spectra of the cross-linking agents were recorded on a Bruker Avance III spectrometer operating at 300 MHz (^1^H) and 75 MHz (^13^C, Attached Proton Test), respectively. For the alginates and kinetic studies, ^1^H NMR spectra were recorded on a Bruker Avance III 400 MHz spectrometer. Kinetic studies were conducted without spinning at ambient temperatures for 2 h (**S1**) and 3 h (**S2** and **S3**) with one spectrum every 190 s. For these experiments, 1 mL 2 wt % solution of alginate peptide conjugate **3** was dissolved in D_2_O at room temperature. 0.5 mL of 1 M phosphate buffer (NaHPO_4_) in D_2_O was added, containing either nothing or glycine (set to a final concentration of 20 mM). To start the enzymatic reaction, 0.5 mL of a papain solution (40 mg mL^–1^ in 1 M phosphate buffer pH 8 in D_2_O, prepared and tempered shortly before) was added. Peptide ester consumption and ethyl alcohol release were measured over time with and without the presence of 20 mM glycine simulating 10 mM 2GlyEDA HCl (**6b**).

### Gel Permeation Chromatography

Polymeric fractions of the alginates were resolved on an UltiMate 3000 HPLC system, equipped with a Phenomenex BioSep-SEC-S 2000 column (300 × 7.8 mm^2^) at 30 °C and a Knauer Smartline 2300 RI detection unit. 0.1 M NaNO_3_ in H_2_O dest. was used as a mobile phase at a flow rate of 0.8 mL min^-1^ for 30 min. Samples were dissolved in the same mobile phase with a concentration of 6 mg mL^–1^ (injection volume: 0.25 mL).

### Time-Dependent Oscillatory Shear Rheology

Change of the viscoelastic properties during the gelation process was monitored by using a modular compact rheometer (MCR 502, Anton Paar, Austria) equipped with a temperable chamber with enabled temperature control around a plate/plate measuring system (*r* = 12.5 mm, *h* = 0.5 mm). Evaporation of the solvent during the measurement was avoided by sealing off the air-exposed surface of the sample with silicon oil. The oil was contained around the sample area with a small gasket. An image of the setup as well as information regarding the drying effect can be viewed in the Supporting Information (Figures S22 and S23). The viscoelastic properties were monitored at a constant frequency of 1 Hz and an oscillating shear strain of 1% for 2–4 h with 1 data point every 10 s.

### Charge Titration

pH potentiometric titrations were performed to determine the quantity of functional groups and their p*K*_a_ values. A two-buret automatic titrator T70 (Mettler Toledo, Switzerland) was used for the titration. The analyte was prepared by first dissolving the synthesis products in 30 mL of ultrapure water, followed by the addition of 3 mol L^–1^ KCL to maintain a constant ionic strength of 0.1 mol L^–1^. The analyte was titrated in a forward (acidic to alkaline) and back (alkaline to acidic) fashion between 2 < pH < 11 using 0.1 mol L^–1^ HCL and KOH as titrants. The forward and back-titration allows one to follow the deprotonation (forward) and protonation (back) processes separately and observe possible ester hydrolysis. The analyte was titrated in an inert atmosphere by purging with nitrogen gas to prevent the CO_2_ dissolution. The pH was recorded using a combined glass electrode DG115 (Mettler Toledo, Switzerland). Detailed data treatment procedure and calculations are described elsewhere.^44,45^ The difference of charge per mass compared to the unmodified **1** (Na-Alg) allows an estimation of the degree of substitution (DS) using eq 1, where *q*_mod_ is the charge per mass of the modified alginate, *M*_Alg_ the molar mass of a monomeric unit of unmodified alginate and *M*_mod_ the molar mass of a modified monomeric unit.^46^

$$\mathrm{DS}=\frac{q_{\mathrm{mod}}M_{\mathrm{Alg}}-1}{q_{\mathrm{mod}}(M_{\mathrm{Alg}}-M_{\mathrm{mod}})-1}$$ 1

## Supplementary Material



## Notes

Synthetic procedure and analytical data of the cross-linking agents; gelation in DMEM cell media; setup for rheological measurements; strain stiffening pseudo-PG nanofiber gels; and results on the papain-catalyzed oligomerization of glycine peptide esters (PDF)
