# Supplementary material for: Chemo-Enzymatic Synthesis of Viscoelastic Pseudopeptidoglycan Networks
Source: Bioconjug Chem. 2025 Jun 24;36(9):1933–42. doi: 10.1021/acs.bioconjchem.5c00014 (PMC12447390; doi:10.1021/acs.bioconjchem.5c00014)
Supplement: Supplementary file 1 [file bc5c00014_si_001.pdf]

Supporting Information

Chemo-enzymatic synthesis of viscoelastic pseudopeptidoglycan networks

Philipp Loibner[1], David Bučak-Gasser[1], Katharina Schober[1], Tobias Steindorfer[1], Monika Brandtner[1], Tobias Dorn[1], Tanja Wrodnigg[1], Dmytro Neshchadin[2], Georg Gescheidt-Demner[2], Matej Bračič[3], Florian Lackner[1], Tamilselvan Mohan[1], Karin Stana Kleinschek[1], Rupert Kargl[1,3]

[1]*Institute of Chemistry and Technology of Biobased Systems, IBIOSYS Graz University of Technology, Austria*
[2]*Institute of Physical and Theoretical Chemistry, Graz University of Technology, Austria*
[3]*Institute of Engineering Materials and Design, University of Maribor, Slovenia*

A Synthetic procedure and analytical data of the crosslinking agents

A.1 NMR spectra

A.1.1 2GlyEDA

Synthesis of bis(glycinamide) ethylenediamine (2GlyEDA HCl)

For the synthesis of the bifunctional crosslinking agent, *N*-Boc-protected glycine (compound **4**, Boc-Gly-OH) was coupled with ethylenediamine **5** via carbodiimide (EDC HCl) activation in THF in a semi-heterogeneous way. Boc-cleavage was performed under acidic conditions in 1,4-dioxane yielding the product as hydrochloride salt (***Figure S1***).

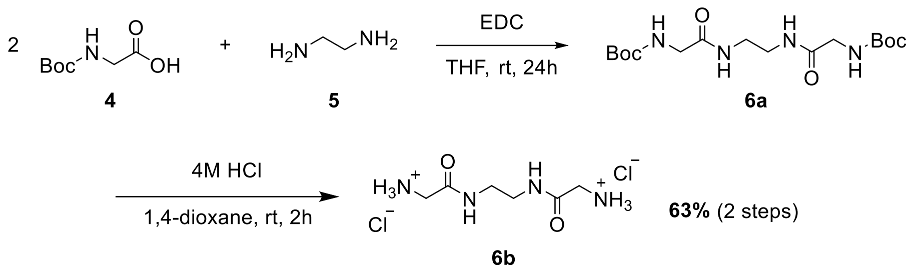

Figure S1: Synthesis of the bifunctional crosslinking agent **6b** (2GlyEDA HCl)

1,12-Bis(1,1-dimethylethyl) 4,9-dioxo-2,5,8,11-tetraazadodecanedioate **6a**

EDC HCl (1.95 g, 11 mmol) was suspended in a solution of 2.0 g (11 mmol) Boc-Gly-OH **4** in 25 ml THF. 0.38 ml (5.7 mmol) EDA **5** were added, and the mixture was stirred at rt for 24 h. Remaining solids were filtered off and the solvent was removed under reduced pressure, which gave the crude product as a colourless oil. Purification by flash chromatography (mobile phase: EtOAc/MeOH 9/1 v/v) gave the diamide as a white solid (1.36 g, yield: 64%). ATR-IR: 3325, 2976, 2931, 1703, 1669, 1540, 1526, 1451, 1423, 1389, 1366, 1282, 1241, 1222, 1158, 1051, 1030 cm^{-1}; ^{1}H NMR (300 MHz, DMSO-d_6): δ= 7.83 (2H, s, NH), 6.90 (2H, NH), 3.51 (4H, d, CH_2), 3.12 (4H, t, CH_2), 1.40 (18H, s, CH_3); ^{13}C NMR APT (75.5 MHz, DMSO-d_6): δ= 169.45 (C), 155.59 (s, C), 78.01 (s, C), 43.25 (s, CH_2), 38.31 (s, CH_2), 28.17 (s, CH_3); EA: calc. C, 51.32; H, 8.08; N, 14.96; found C, 51.14; H, 7.98; N, 14.54.

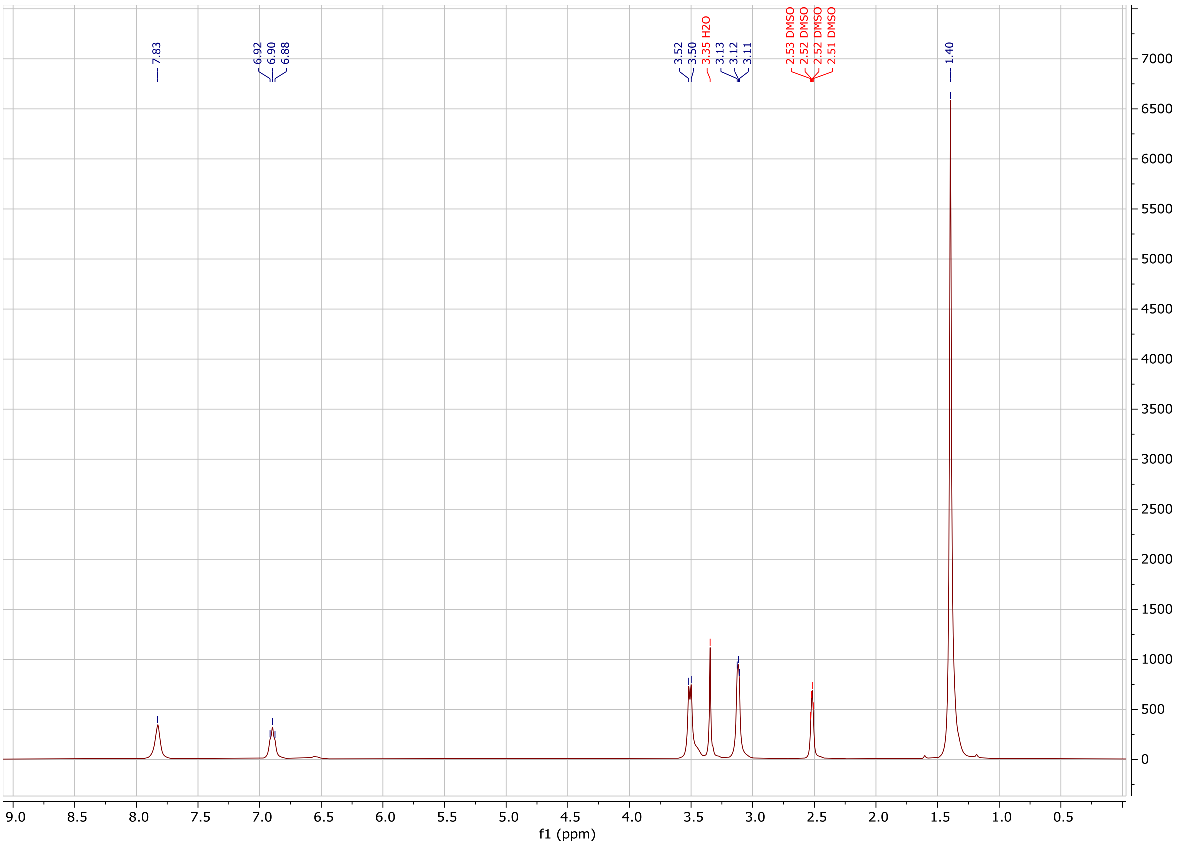

Figure S2: ^{1}H-NMR spectrum of compound **6a**

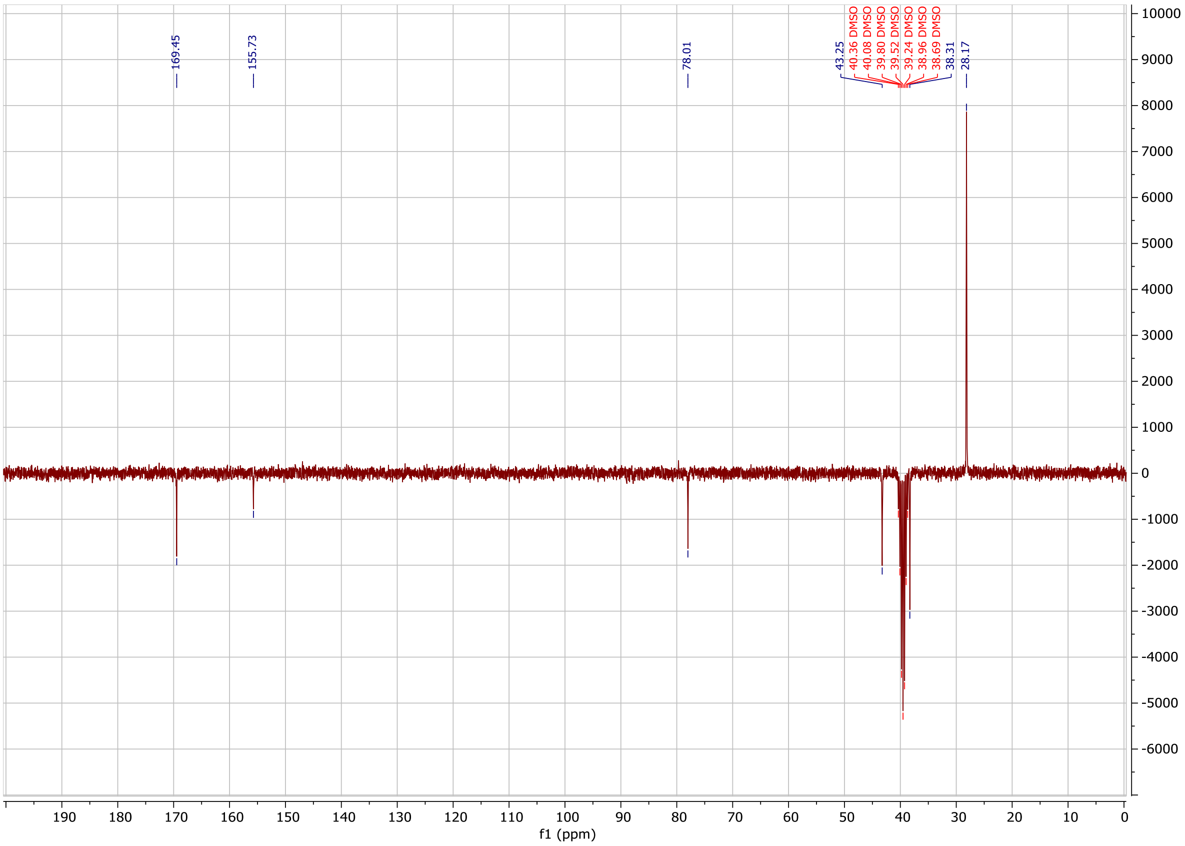

Figure S3: ^{13}C-NMR (APT) spectrum of compound **6a**

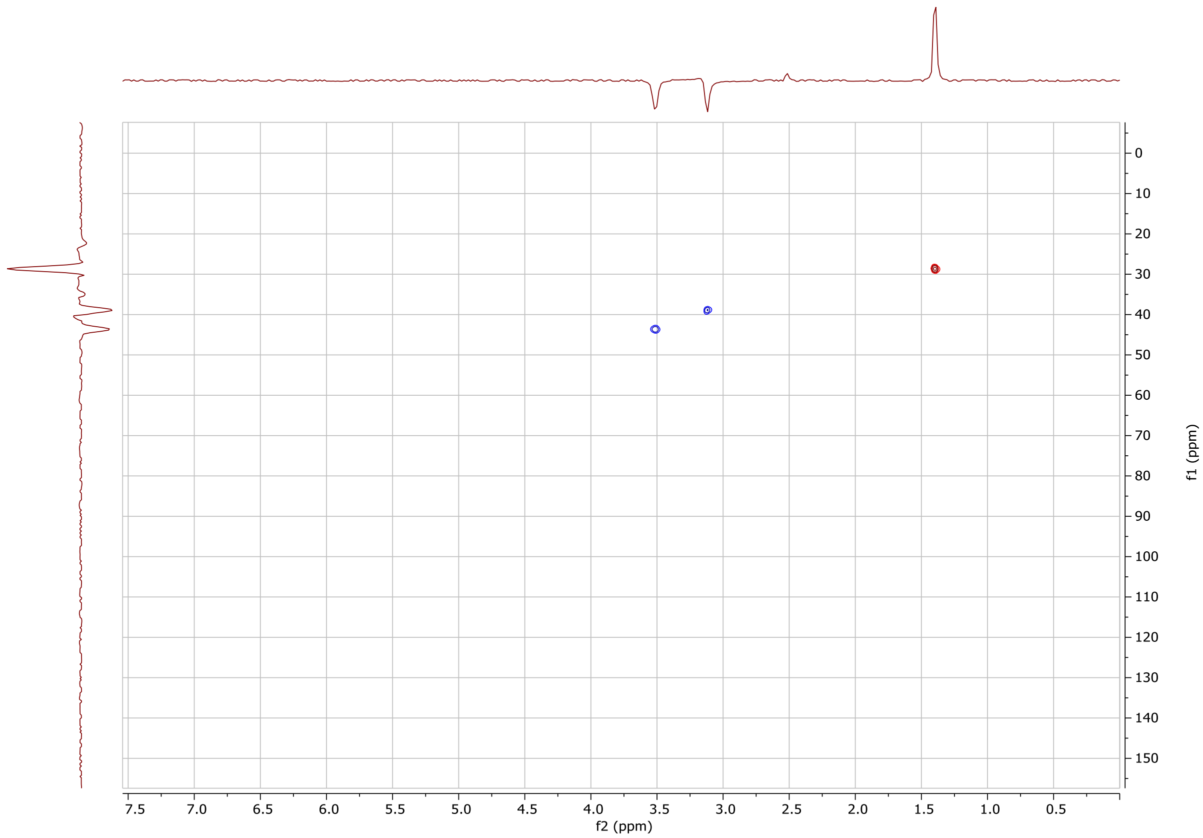

Figure S4: HSQC spectrum of compound ***6a***

N,N'-1,2-Ethanediylbis[2-aminoacetamide] dihydrochloride **6b**

0.22 g (0.6 mmol) of compound **6a** were suspended in 20 ml 4M HCl in 1,4-dioxane and stirred for 2 h at rt. Removal of the solvent under reduced pressure gave compound **6b** (2GlyEDA) as a light brown solid (0.15 g, yield: 98%). ATR-IR: 3198, 3099, 3069, 3011, 2919, 2870, 2681, 2610, 2488, 2412, 1652, 1604, 1567, 1506, 1460, 1433, 1386, 1322, 1271, 1231, 1112, 1066, 1005 cm^{-1}; ^{1}H NMR (300 MHz, DMSO-d_6): δ= 8.74 (2H, t, NH), 8.29 (6H, s, NH_3^+), 3.54 (4H, s, CH_2), 3.21 (4H, t, CH_2); ^{13}C NMR APT (75.5 MHz, DMSO-d_6): δ= 166.08 (s, C), 40.20 (s, CH_2), 38.19 (s, CH_2); EA: calc. C, 29.16; H, 6.53; N, 22.67; found C, 33.14; H, 6.86; N, 20.22.

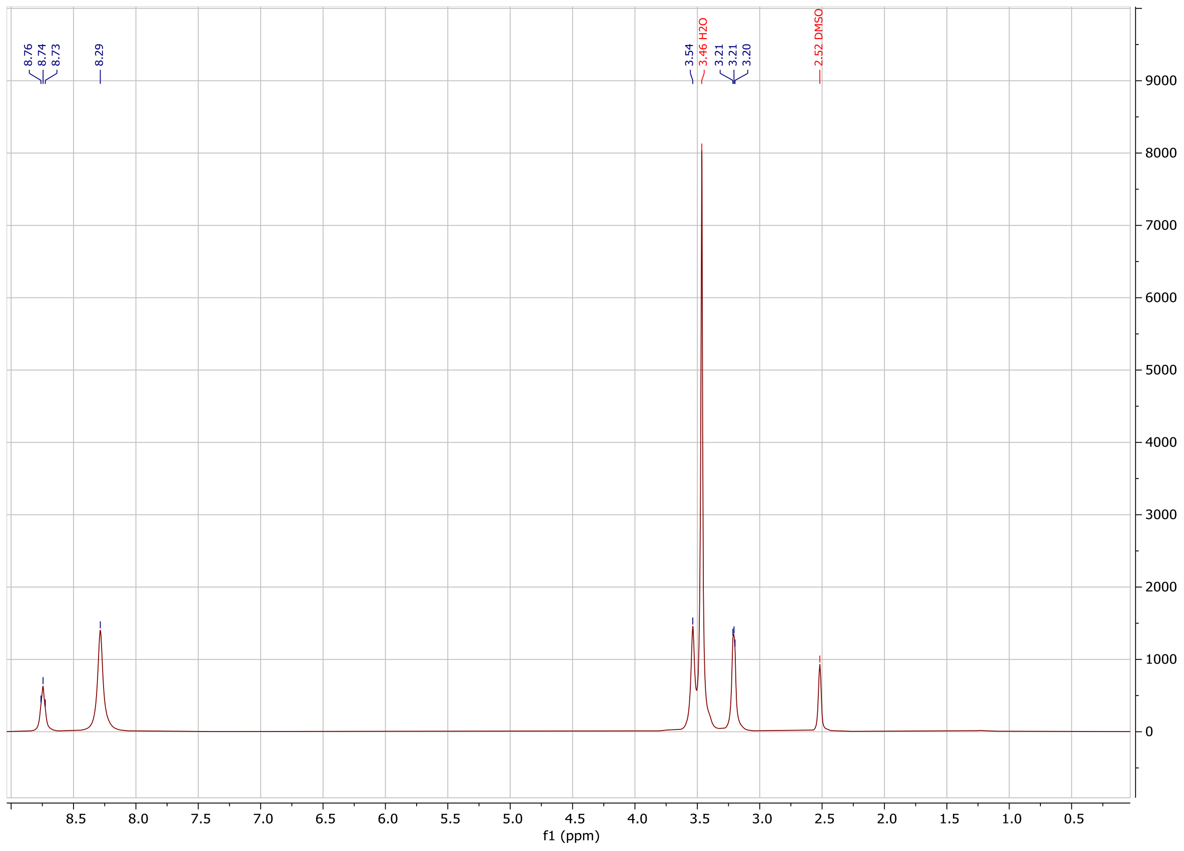

Figure S5: ^{1}H-NMR spectrum of compound **6b**

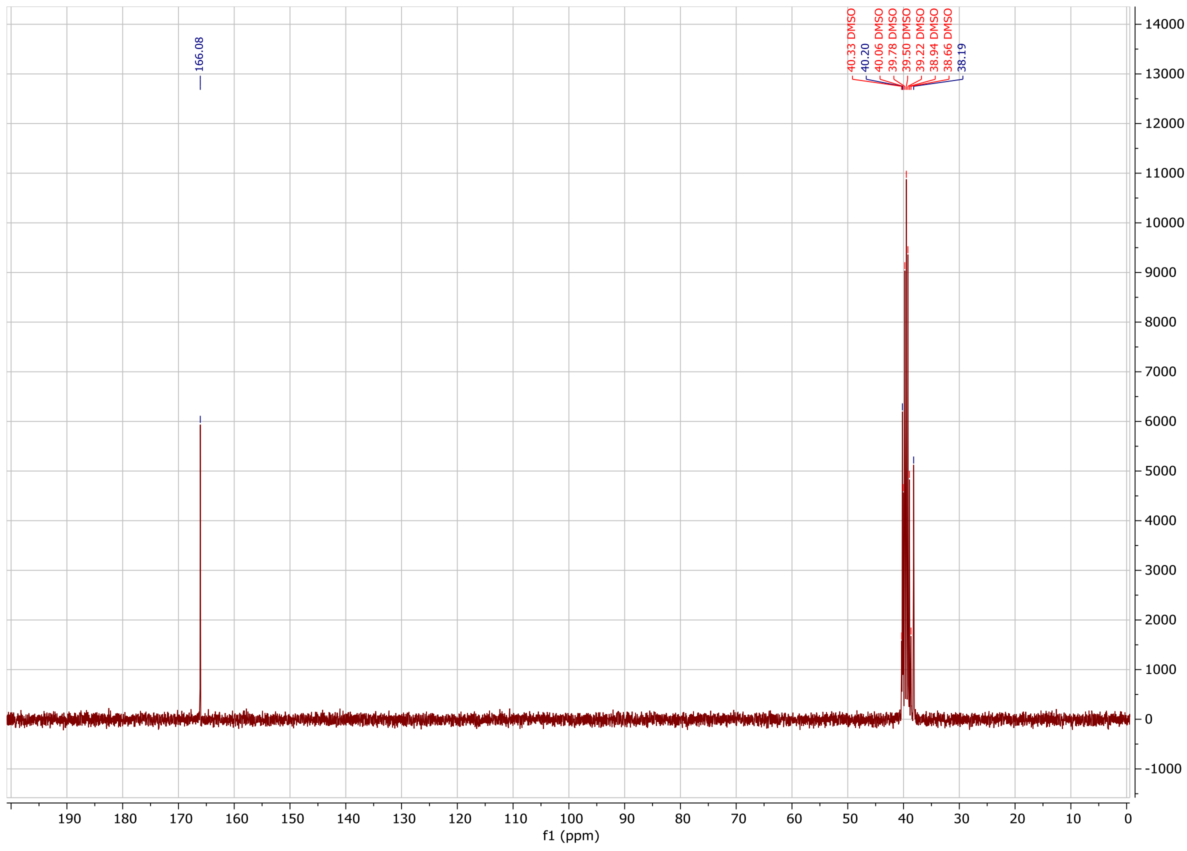

Figure S6: ^{13}C-NMR (APT) spectrum of compound **6b**

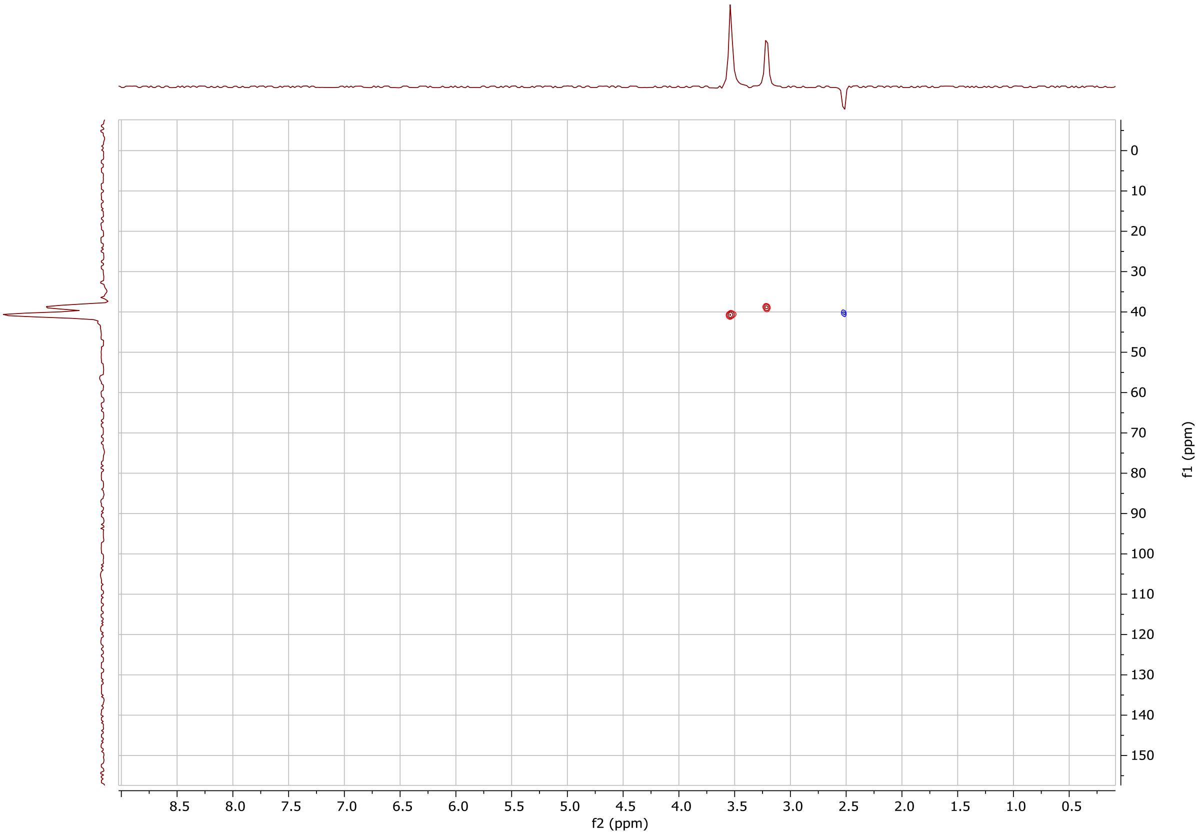

*Figure S7: HSQC spectrum of compound **6b***

A.1.2 *4GlyPE*

Synthesis of pentaerythritol tetrakis(glycinate) (4GlyPE HCl)

The tetrafunctional crosslinking agent was synthesized by esterification of **7** (pentaerythritol) with four equivalents of **4** (Boc-Gly-OH), at elevated temperature in DMSO using carbonyldiimidazole as an activating agent (***Figure S8***). Deprotection was performed analogously to 2GlyEDA HCl.

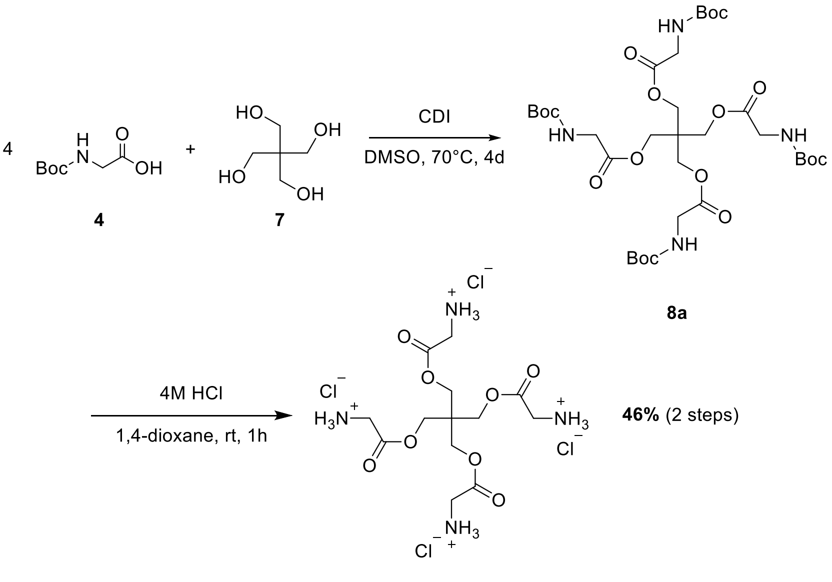

Figure S8: Synthesis of the tetrafunctional crosslinking agent **8b** *(4GlyPE* HCl)

3-([[[(tert-butoxycarbonyl)amino]acetyl}oxy)-2,2-bis[([[(tert-butoxycarbonyl)amino]acetyl}oxy)methyl]propyl [(tert-butoxycarbonyl)amino]acetate **8a**

5.25 g (30 mmol) Boc-Gly-OH **4** were dissolved in 50 ml DMSO and heated to 70 °C via oil bath. CDI (4.86 g, 30 mmol) was added in portions and the mixture was left stirring for 2 h. Then, a solution of 0.68 g (5 mmol) PE **7** in 50 ml DMSO was added and the mixture was left to react for 4 d at 70 °C. The reaction was cooled to rt and extracted by adding 50ml EtOAc and 100 ml H_2O. The aqueous phase was washed with 50 ml EtOAc (3x) and the combined organic phases with 50 ml H_2O (3x) followed by 50 ml brine (1x). The organic layer was dried over Na_2SO_4 and the solvent was removed under reduced pressure giving the crude product mixture as a yellow oil. Purification by flash chromatography (mobile phases: EtOAc/Cyclohexane 1/3, 1/2, 1/1 and 2/1 v/v) gave compound **8a** as a white amorphous foam, which solidified overnight (1.90 g, yield: 50%). ATR-IR: 3377, 3012, 2977, 2935, 2905, 2874, 1770, 1759, 1698, 1515, 1475, 1457, 1407, 1392, 1366, 1295, 1281, 1251, 1158, 1141, 1057, 1036 cm^{-1}; ^{1}H NMR (300 MHz, DMSO-d_6): δ= 7.02 (4H, t, NH), 3.91 (8H, s, CH_2), 3.48 (8H, d, CH_2), 1.16 (36H, s, CH_3); ^{13}C NMR APT (75.5 MHz, DMSO-d_6): δ= 169.37 (s, C), 155.86 (s, C), 78.43 (s, C), 62.54 (s, CH_2), 41.80 (s, CH_2), 28.06 (s, CH_3); EA: calc. C, 51.82; H, 7.38; N, 7.33; found C, 51.89; H, 7.48; N, 7.25.

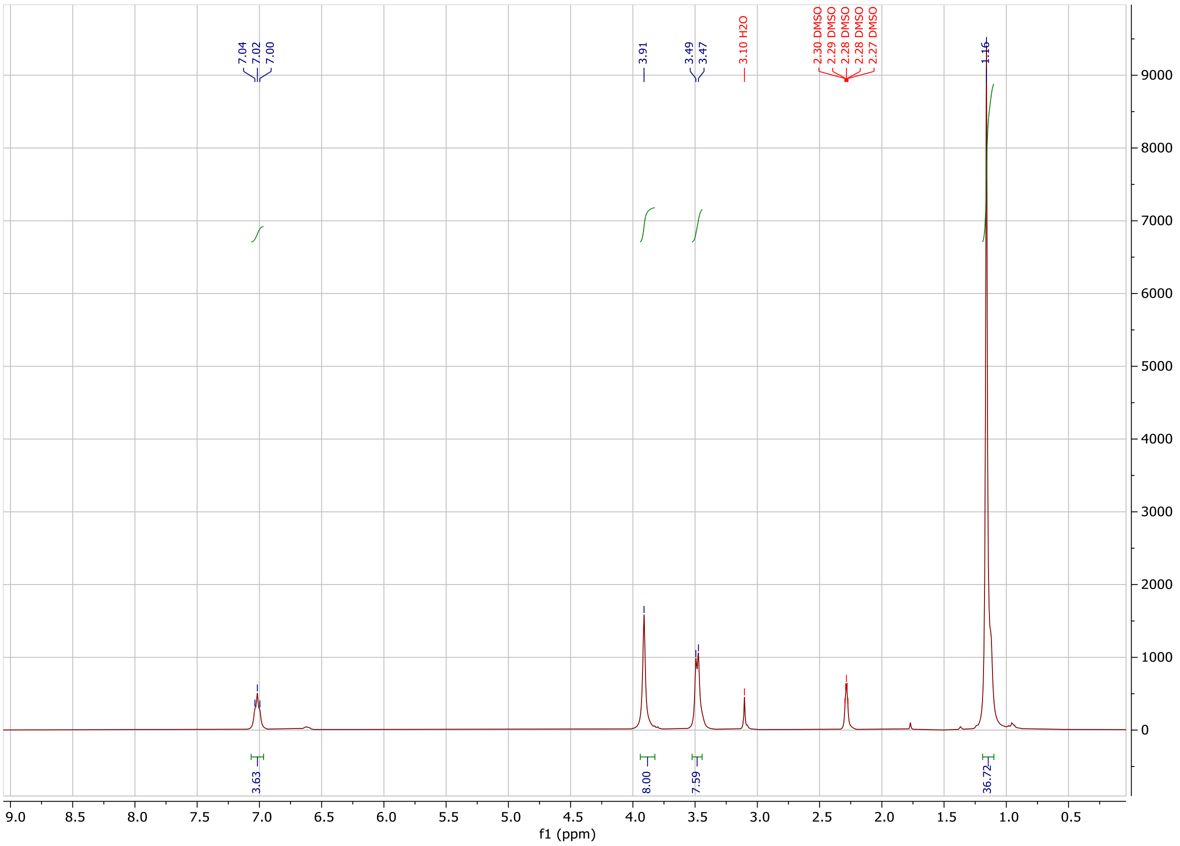

*Figure S9: ^{1}H-NMR spectrum of compound **8a***

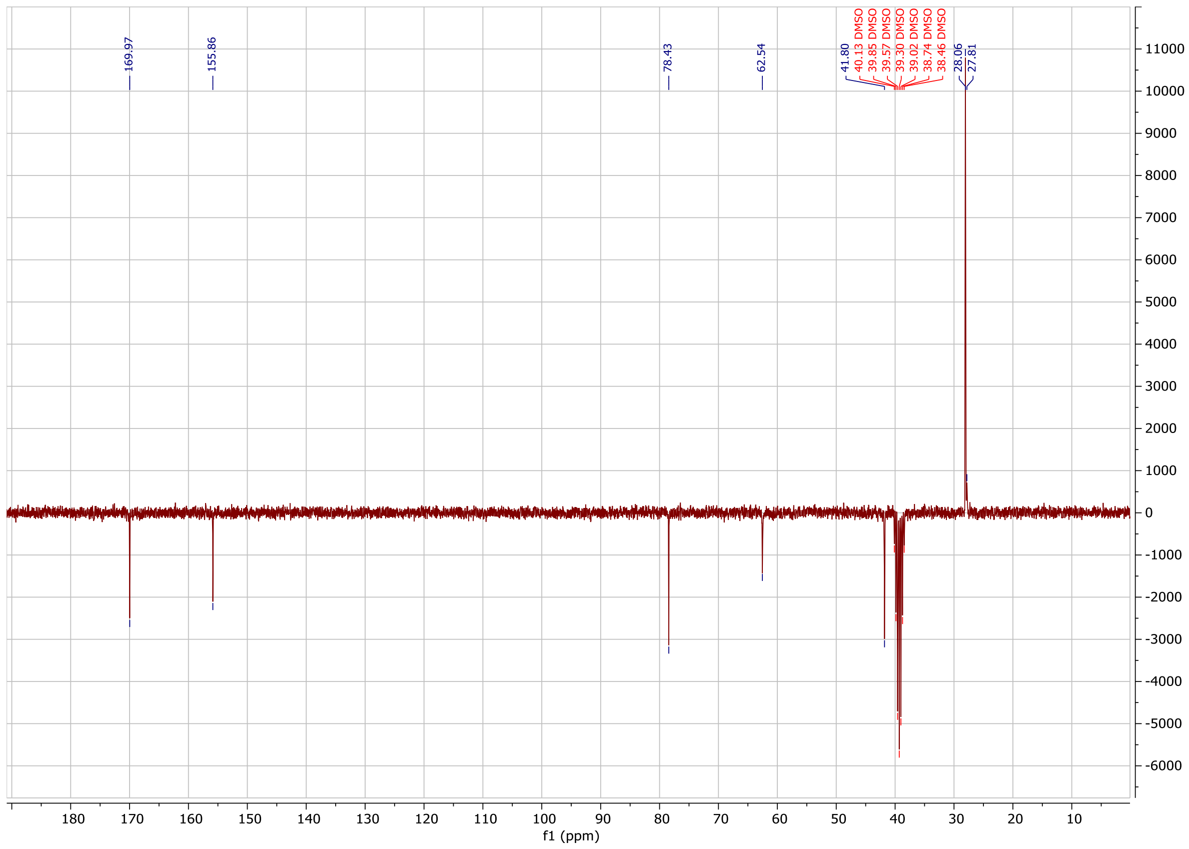

*Figure S10: ^{13}C-NMR (APT) spectrum of compound **8a***

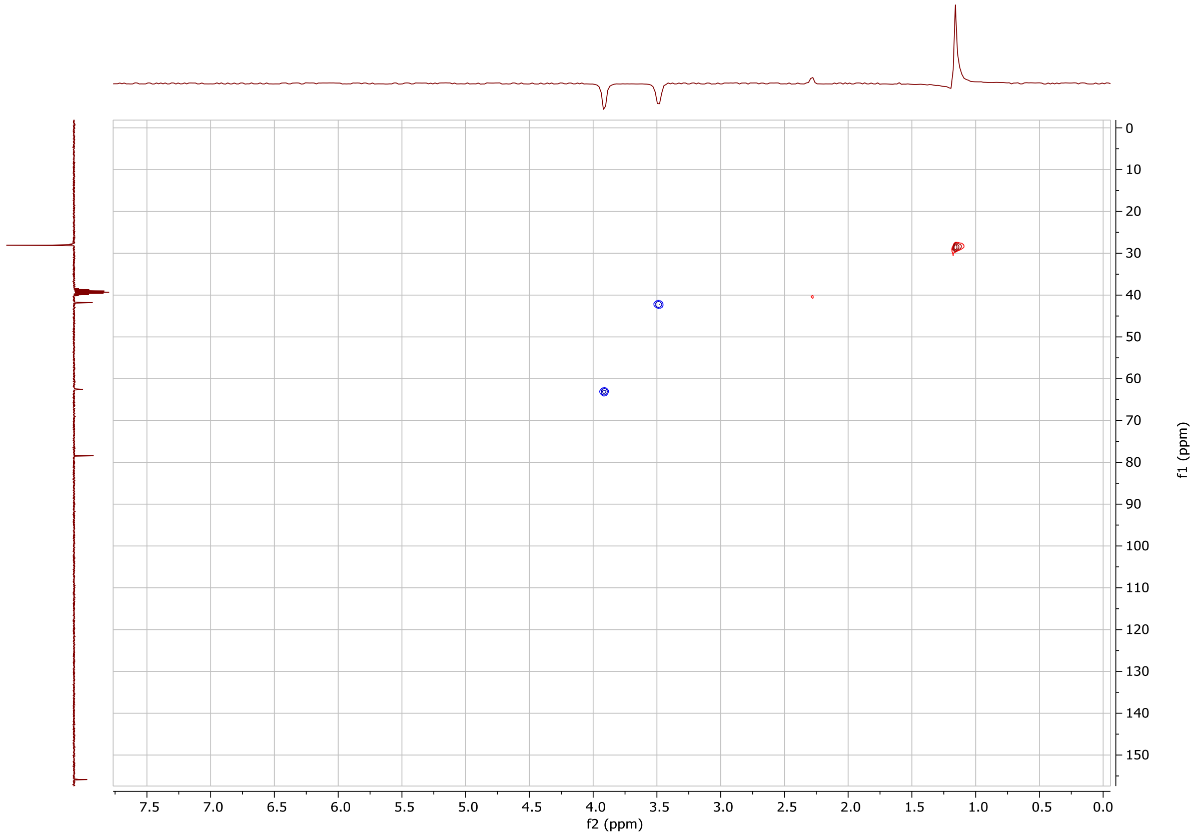

*Figure S11: HSQC spectrum of compound **8a***

3-([aminoacetyl}oxy)-2,2-bis[([aminoacetyl}oxy)methyl]propyl aminoacetate tetrahydrochloride **8b**

0.45 g (0.6 mmol) of compound **8a** were dissolved in 20 ml 4 M HCl in 1,4-dioxane and stirred for 1 h at rt. Removal of the solvent under reduced pressure gave compound **8b** (4GlyPE) as a light brown solid (0.33 g, yield: 92%). ATR-IR: 3401, 2956, 2863, 2704, 2617, 1746, 1587, 1490, 1413, 1377, 1311, 1224, 1112, 1049 cm^{-1}; ^{1}H NMR (300 MHz, DMSO-d_6): δ= 8.72 (12H, s, NH_3^+), 4.33 (8H, s, CH_2), 3.83 (8H, s, CH_2); ^{13}C NMR APT (75.5 MHz, DMSO-d_6): δ= 166.87 (s, C), 62.85 (s, CH_2), 41.82 (s, C), 39.70 (s, CH_2); EA: calc. C, 30.61; H, 5.53; N, 10.98; found C, 29.77; H, 5.91; N, 9.92.

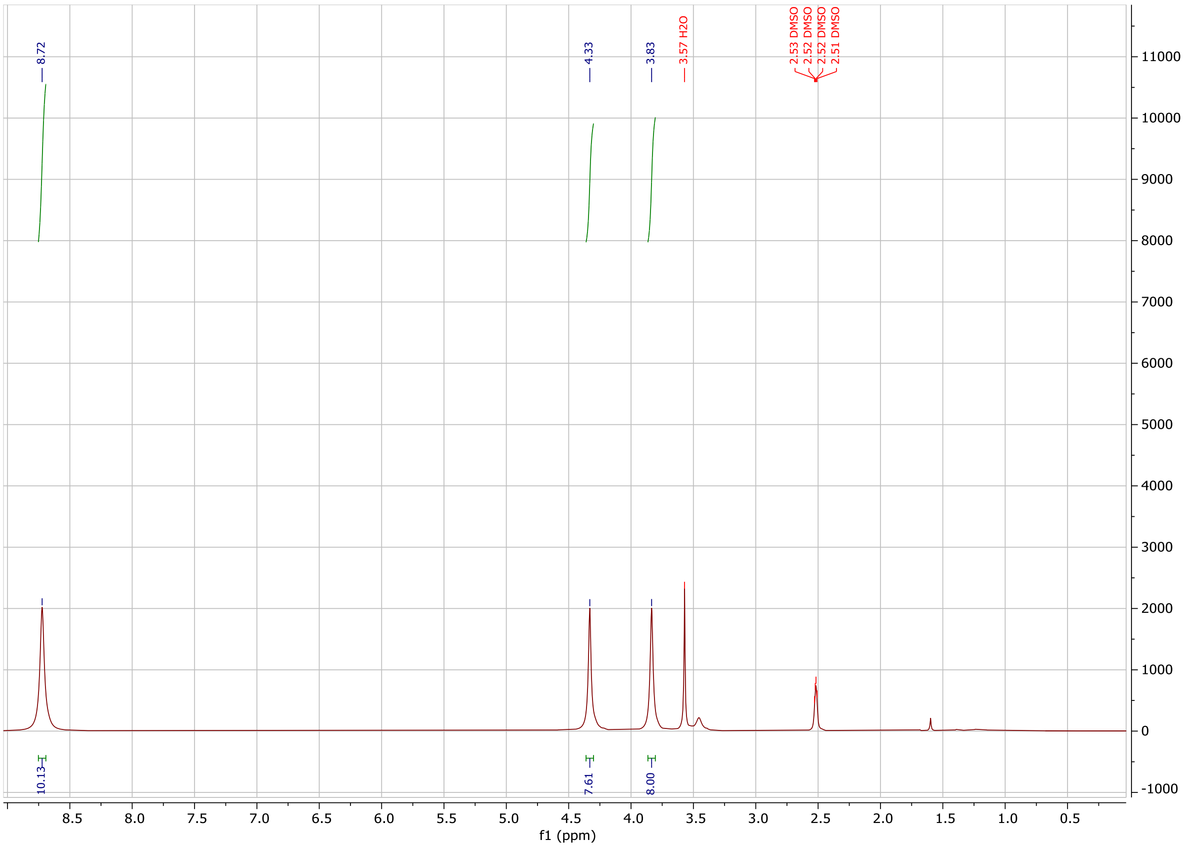

Figure S12: ^{1}H-NMR spectrum of compound ***8b***

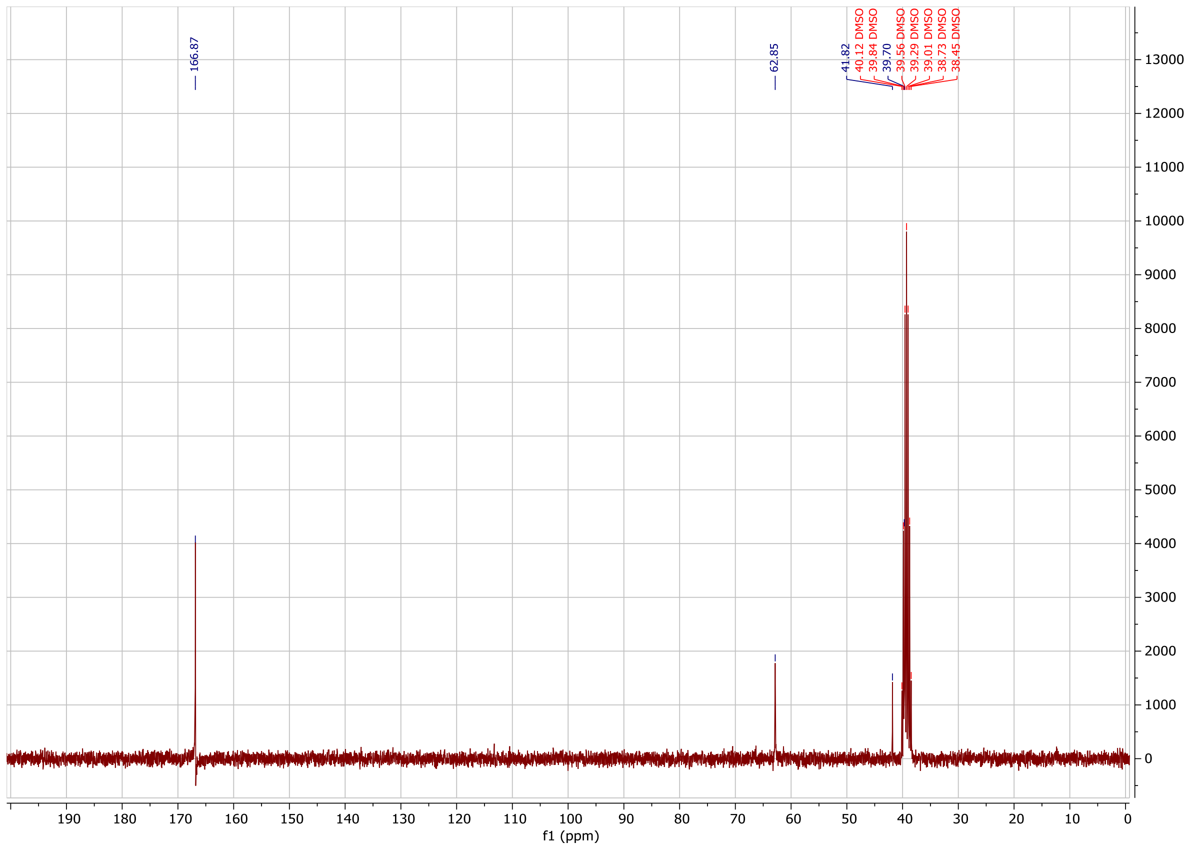

*Figure S13: ^{13}C-NMR (APT) spectrum of compound **8b***

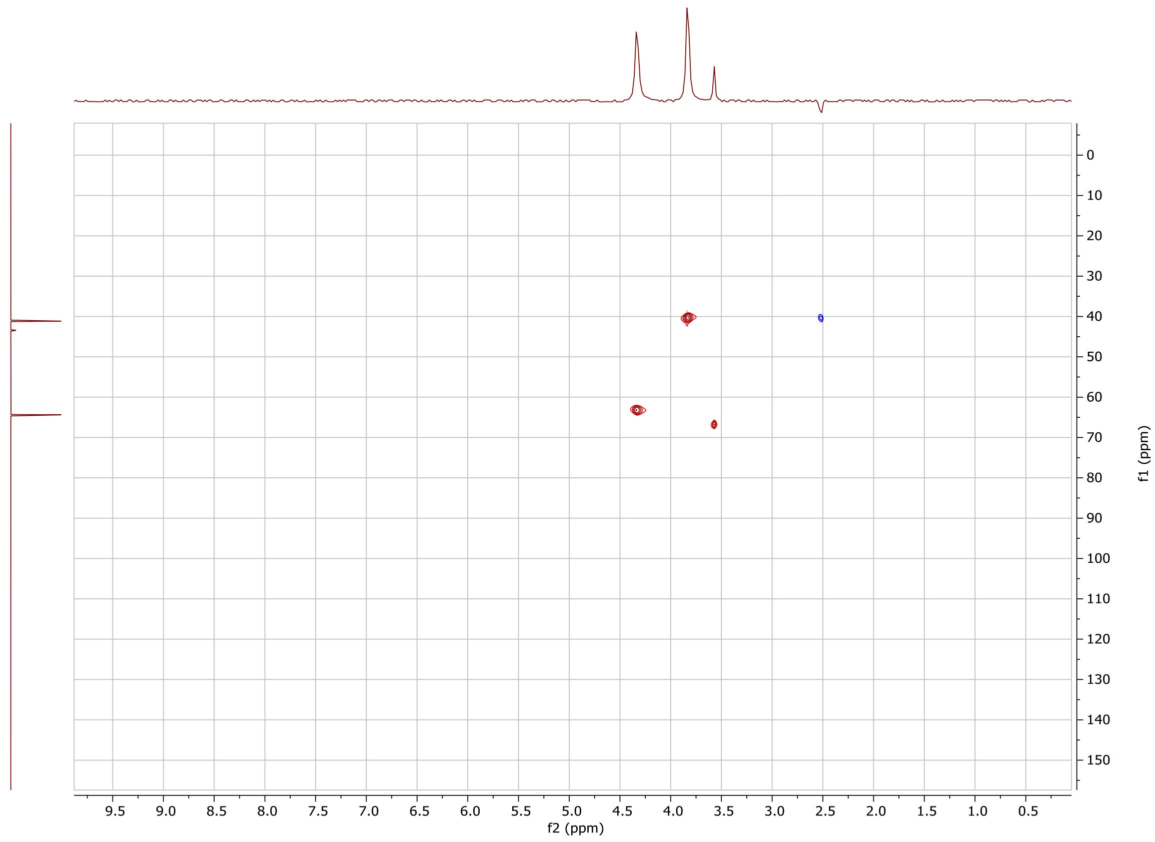

*Figure S14: HSQC spectrum of compound **8b***

A.2 ATR-IR spectra

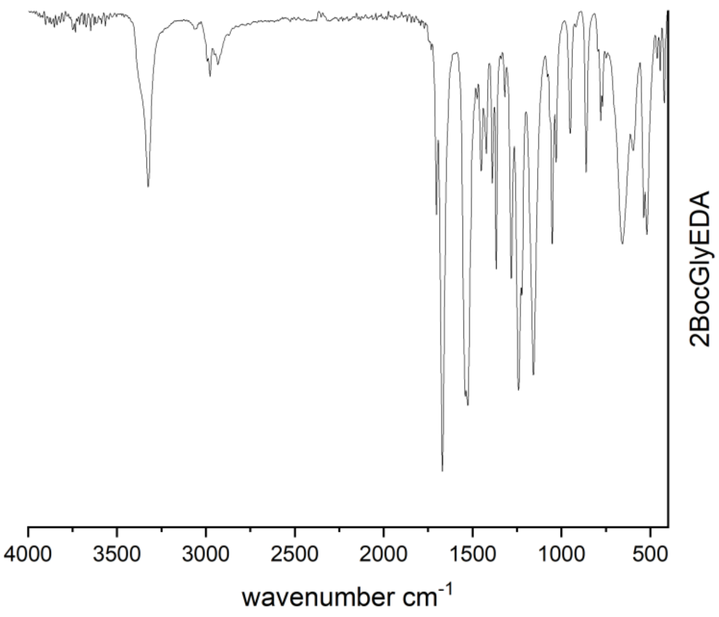

*Figure S15: ATR-IR spectrum of compound **6a***

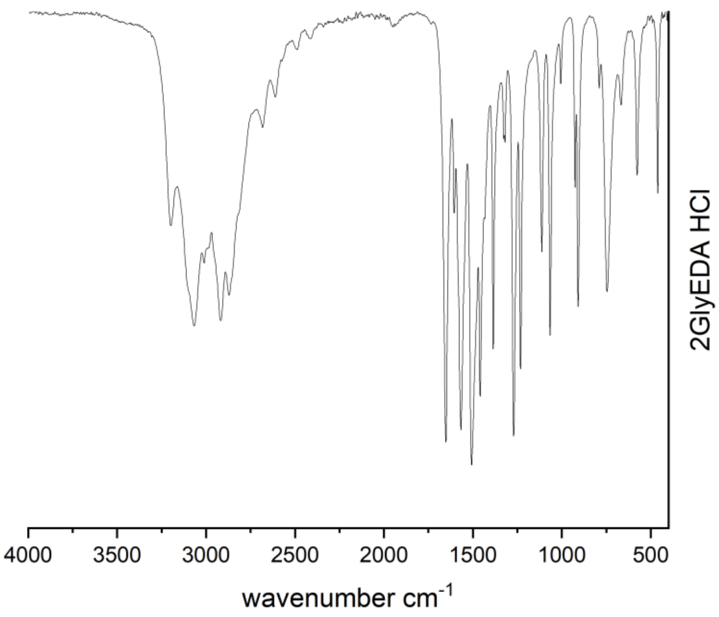

*Figure S16: ATR-IR spectrum of compound **6b***

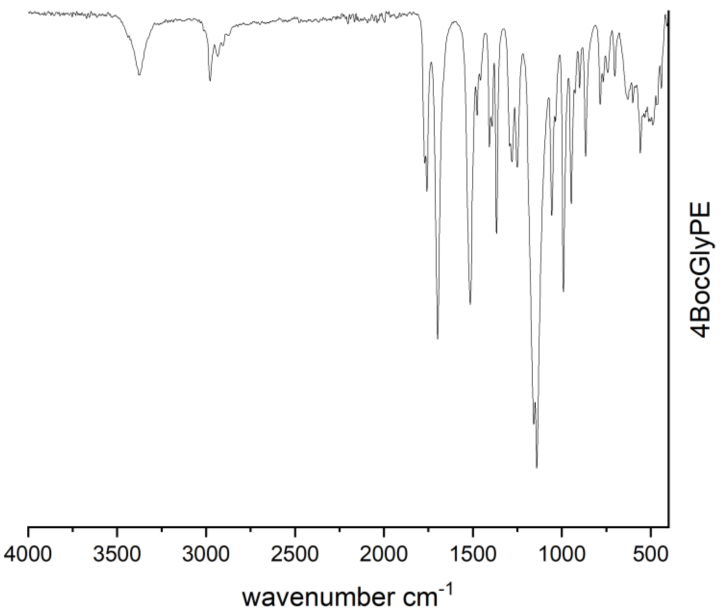

Figure S17: ATR-IR spectrum of compound ***8a***

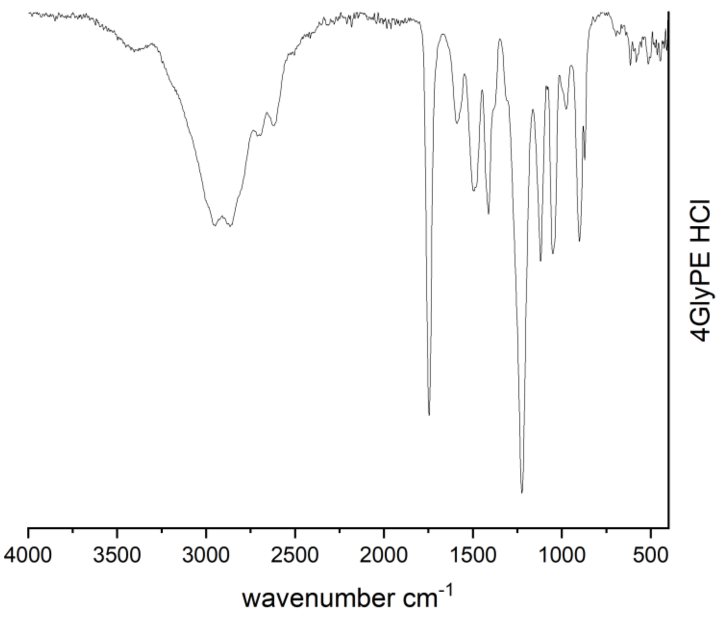

Figure S18: ATR-IR spectrum of compound ***8b***

A.3 Elemental analysis

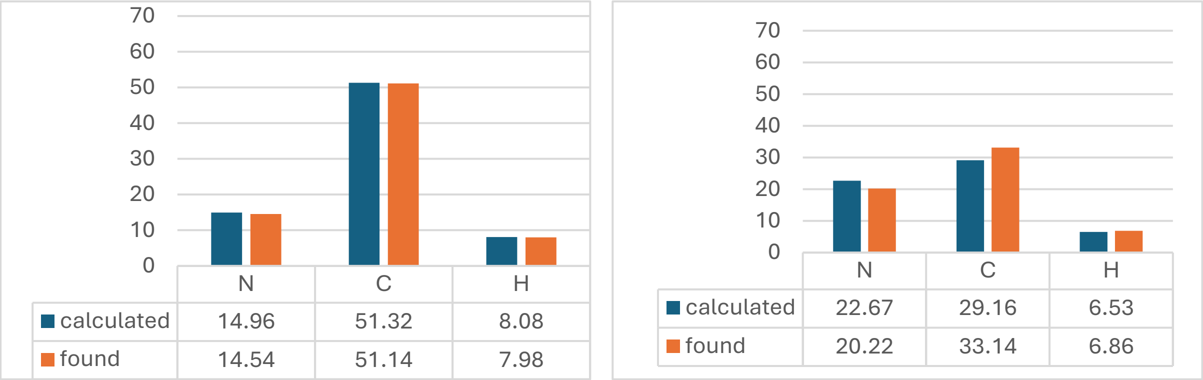

	N	C	H
calculated	14.96	51.32	8.08
found	14.54	51.14	7.98

	N	C	H
calculated	22.67	29.16	6.53
found	20.22	33.14	6.86

Figure S19: Elemental composition of compound ***6a*** *(left) and compound* ***6b*** *(right)*

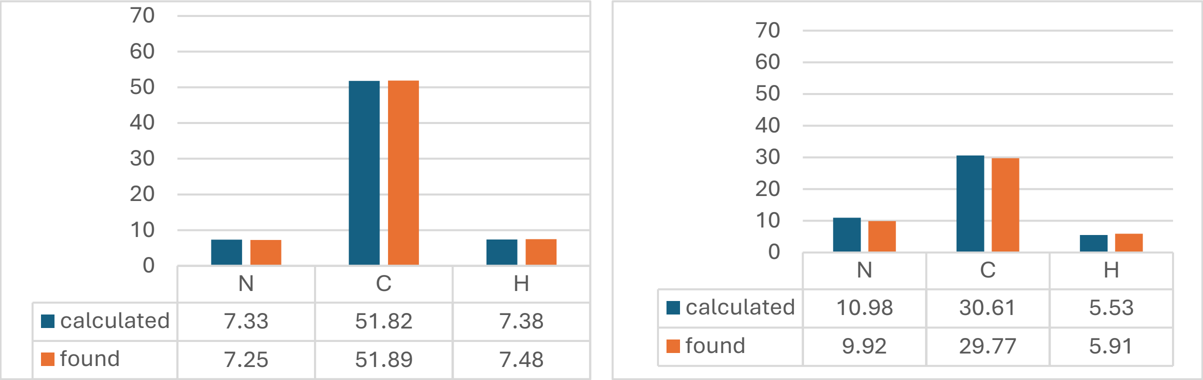

	N	C	H
calculated	7.33	51.82	7.38
found	7.25	51.89	7.48

	N	C	H
calculated	10.98	30.61	5.53
found	9.92	29.77	5.91

Figure S20: Elemental composition of compound ***8a*** *(left) and compound* ***8b*** *(right)*

B Gelation in DMEM cell media

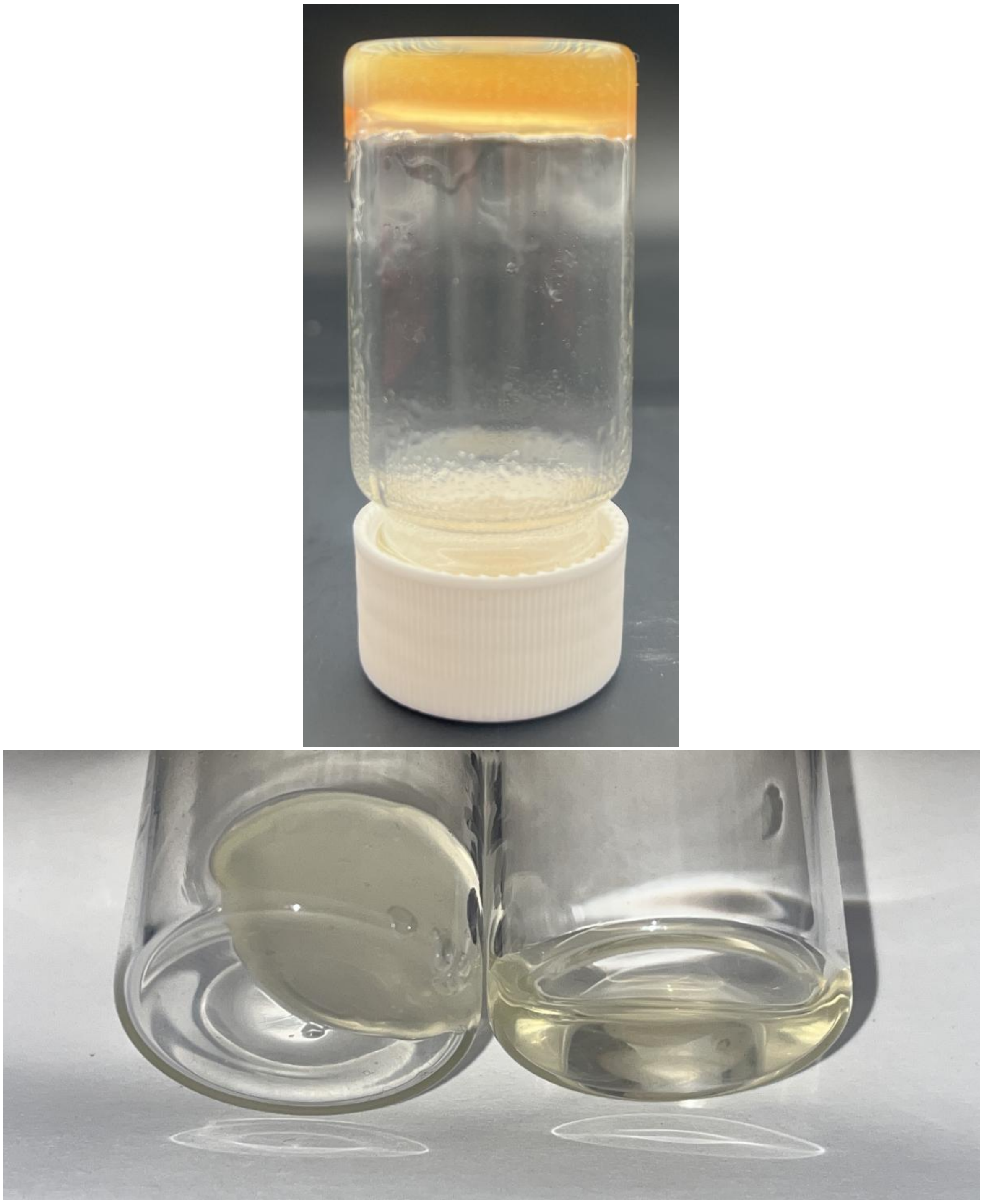

Figure S21: Image of Alg-GlyglyglyOEt before (bottom right) and after enzymatic gelling (bottom left)

C Setup for rheological measurements

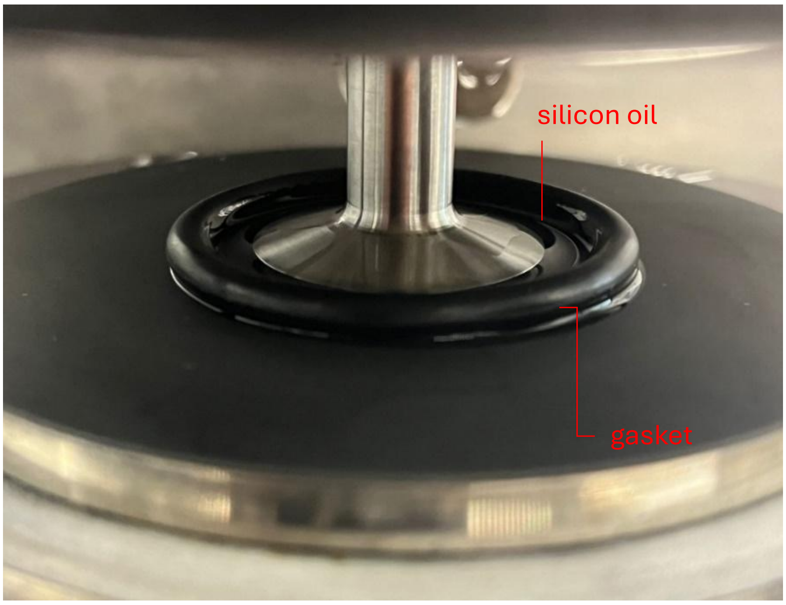

Figure S22: Setup for the time-dependent oscillatory rheology measurements

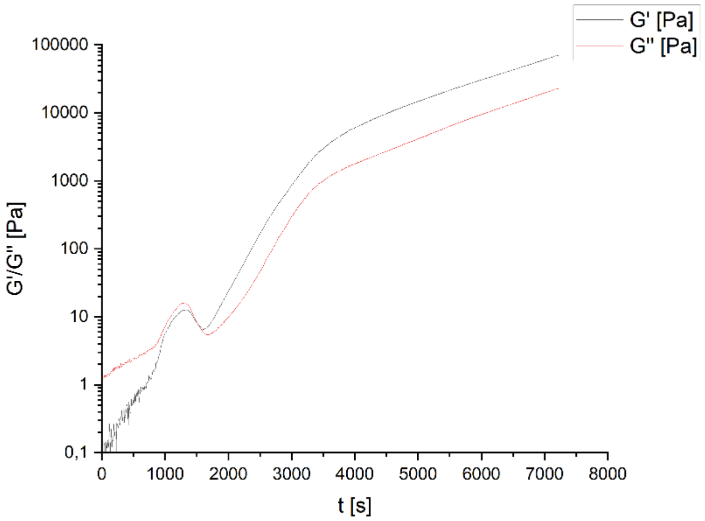

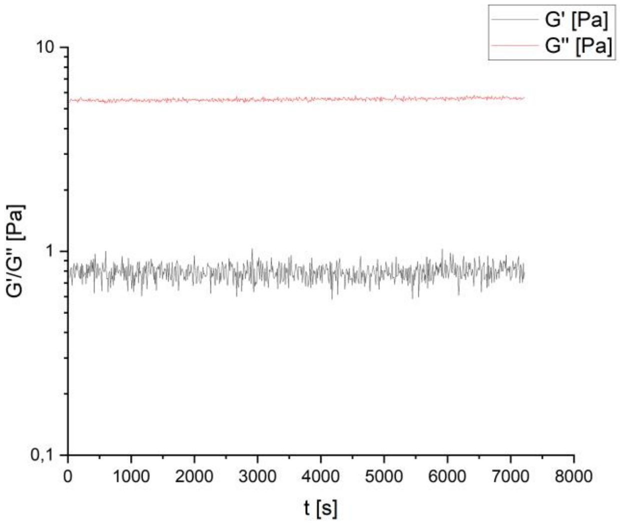

Figure S23: Time-dependent oscillatory shear rheology measurements of Na-Alg solutions (2w%) at 40°C without (left) and with the described setup (right); sealing off the air-exposed surface stabilizes viscoelastic properties during the measurement

D Strain stiffening pseudo-PG nanofiber gels

Bioink preparation for mechanical tests

0.3 g of finely crushed Alg-GlyGlyGlyOEt **3** was mixed with 4.5 g NFC suspension (3 w%) by using a mechanical laboratory stirrer (RZR 2005, Heidolph, Germany) equipped with a laboratory spatula (2000 rpm for approx. 30 min). Then, phosphate salts were added to the mixture (0.5 M and pH 8 in the final ink), followed by 0.037 g of 2GlyEDA HCl **6b**. The compounds were mixed for another 10 min at 2000 rpm. The homogeneous mixture as well as a papain solution (0.3 g/ml in 0.5 M phosphate buffer pH 8) were put on an ice bath and were left to cool for 5 min. Finally, 0.5 ml of the pre-cooled Papain solution was added and mixed thoroughly on the ice bath at 500 rpm. For the second ink with higher NFC content, half the amount of Alg-GlyGlyGlyOEt, 2GlyEDA HCl and papain were mixed into 5.0 g of NFC suspension and treated analogously. **Table S1** gives an overview of the final compositions.

Table S1: Composition of the bioink used for mechanical testing

Ink	NFC	Alg-GlyGlyGlyOEt	2GlyEDA HCl	Papain	Phosphate buffer (0.5 M, pH 8)
**1**	2.3 w%	5.7 w%	30 mM	30 mg/ml	5 ml
**2**	2.9 w%	2.9 w%	15 mM	15 mg/ml	5 ml

Tensile Tests

For evaluation of the mechanical properties of the crosslinked material, dog-bone shaped specimens (50 mm × 8.5 mm × 2mm; according to DIN 53504 S3A) were produced by casting the cooled bioink into negative shapes made from a silicone elastomer (*Supplementary Information*; *Figure D1*) via a 5 ml syringe equipped with a narrow nozzle. The filled shapes were put into Petri dishes together with water saturated sponges and the material was crosslinked in an oven at 40 °C for 2 h. Uniaxial tensile tests were performed using a Shimanzu AGS-X (Japan) universal testing machine with a speed of 50 mm min^{-1} and standard clamps. Four specimens were tested.

Materials to produce dog-bone shaped specimens

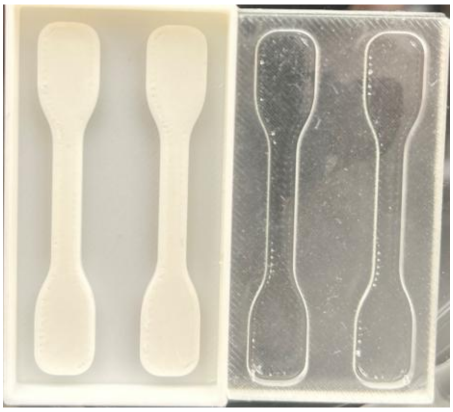

Figure S24: Negative shapes of the used specimens (right) made by casting liquid silicon elastomer in 3D-printed shapes (left) and cross-linking at 50 °C overnight

Preparation and tensile measurements

To observe the papain crosslinked hydrogel in its operative environment, a mixture containing 6w% or 3w% peptide conjugated alginate **3**, 30 mM 2GlyEDA HCl, and 30 mg/ml papain were prepared in a suspension of 3w% nanofibrillated cellulose (NFC) in water. The mixture was crosslinked for 2 h at 40 °C. Mechanical properties of the biomaterial were evaluated via uniaxial tensile tests of dog-bone shaped specimen (n=4). The material reached an average maximal stress of 0.11 MPa (± 7.2%) before breaking at a medial strain of 26% (at 50 mm min^{-1}). ***Figure S25*** shows that the material has intrinsic strain-stiffening behavior as observed in various examples of biological tissue (**Table S2**). Non-linear stress-strain curves can be described via the tangent moduli at initial (E_0 = Young's modulus, in MPa) and terminal strain regions (E_1 in MPa). Furthermore, the x component of the crossing point of the two tangent lines measures the onset of strain-stiffening (critical strain λ_C in m/m) and their ratio $K=E_1/E_0$ is a metric for the strain-stiffening capability.[1] Strain-stiffening arises from the formation of a branched and densely crosslinked filamentous network, as observed in various natural biopolymers such as covalently crosslinked collagen or actin.[2] Geometric interpretations can be seen in ***Figure S25*; left** and the obtained values for the described biomaterial are listed in Table 5.

A gel containing 3w% alginate peptide conjugate **3,** shows a linear stress-strain behavior with a Young's modulus E of 0.024 MPa and a maximal stress of 0.02 MPa (± 8.0%) with an increased medial terminal strain of 79% (***Figure S25*; right**). Note that the obtained values may be underestimated due to elongation of the sample during the measurement, which causes a gradual decrease of the specimen's cross-section area. Using half of the amount of alginate reduces the maximum endurable stress to around 20%. Following the observed trend, higher alginate concentrations may increase the material's durability drastically with the cost of reducing the shear-thinning capability. The full potential of the material, however, can presumably be determined by varying other parameters as well as using a multifunctional crosslinking agent with high hydrolytic stability.

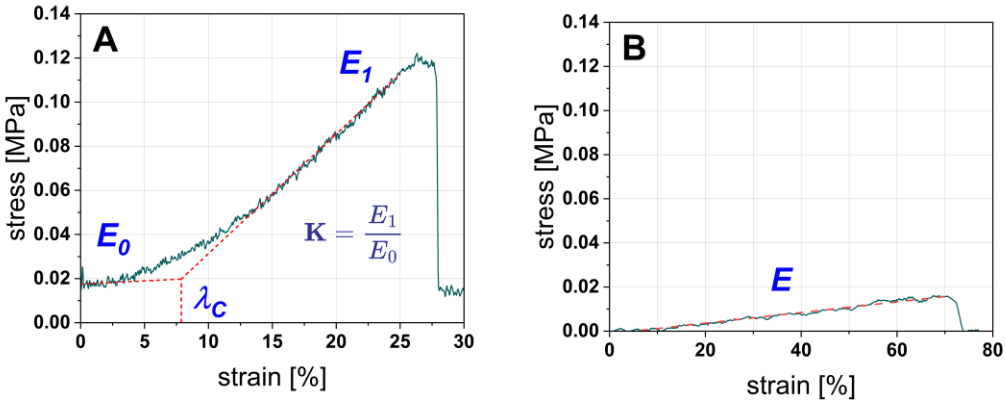

Figure S25: Stress-strain curves of the papain crosslinked biomaterials; **A**) containing 6w% alginate peptide conjugate **3**, with parameters quantifying the strain-stiffening behaviour; **B)** containing 3w% alginate peptide conjugate **3**

Table S2: Strain-stiffening parameters of the papain crosslinked biomaterial containing 6 w% Alg-GlyGlyGlyOEt

E_0 [MPa]	E_1 [MPa]	λ_C [1]	K [1]
0.029	0.542	0.08	18.7

E Papain catalysed oligomerization of glycine peptide esters

The respective substrates **(Table S3)** were dissolved in 5 ml 1 M phosphate buffer (pH 8) and the solutions heated to 40 °C. 50 mg of papain was added and the mixture was left to react for 24 h. The obtained solids were isolated by centrifugation for 5 min at 15000 rpm after transferring into 1.5 ml Eppendorf tubes. The oligomers were washed by addition of 1 ml H_2O dest., mixing on a vortex, centrifugation (5 min, 15000 rpm) and removal of the excess aqueous solution (3x). Finally, the purified products were dried under reduced pressure and weighted.

Table S3: Papain facilitated oligomerization of different amino acid- or peptide-derivatives. Conditions: 1 M phosphate buffer pH 8, papain concentration 10 mg/ml, 40 °C, substrate concentration 2 w%.

substrates	isolable solids [%]
**2w% GlyOMe HCl**	4.8
**2w% GlyOEt HCl**	19.2
**2w% GlyGlyOMe HCl**	19.8
**2w% GlyGlyGlyOEt HCl**	76.2
**2w% GlyGlyGly**	-

1. Xu, J.; Jiang, Y.; Gao, L., Synthetic strain-stiffening hydrogels towards mechanical adaptability. *Journal of Materials Chemistry B* **2023,** *11* (2), 221-243.

2. Burla, F.; Mulla, Y.; Vos, B. E.; Aufderhorst-Roberts, A.; Koenderink, G. H., From mechanical resilience to active material properties in biopolymer networks. *Nature Reviews Physics* **2019,** *1* (4), 249-263.
